# Development and Characterization of a *Zanthoxylum limonella* Essential Oil Nanoemulsion for Oral Care Applications

**DOI:** 10.3390/molecules31213905

**Published:** 2026-10-29

**Authors:** Nisakorn Saewan, Tawanun Sripisut, Maria Pilar Vinardell

**Affiliations:** 1 School of Cosmetic Science, Mae Fah Luang University, 333, Moo.1, Thasud, Muang, Chiang Rai 57100, Thailand; tawanun.sri@mfu.ac.th; 2 Cosmetic and Beauty Innovations for Sustainable Development (CBIS) Research Group, Mae Fah Luang University, Chiang Rai 57100, Thailand; 3 Cosmetic for Beauty and Wellness (CoBeW) Research Group, Mae Fah Luang University, Chiang Rai 57100, Thailand; 4 Departament de Bioquimica i Fisiologia, Facultat de Farmàcia i Ciències de l’Alimentació, Universitat de Barcelona, 08028 Barcelona, Spain

**Keywords:** *Zanthoxylum limonella*, essential oil, nanoemulsion, oral care, storage stability, limonene equivalent retention

## Abstract

*Zanthoxylum limonella* is an aromatic medicinal plant widely used in Thai traditional medicine and oral healthcare. This study aimed to characterize the chemical composition of *Z. limonella* essential oil (ZLO) extracted from fresh and dried fruits and to develop a nanoemulsion to enhance its stability and applicability in oral care products. Essential oils from fresh and shade-dried fruits were obtained by hydrodistillation and analyzed using gas chromatography–mass spectrometry (GC–MS). The major volatile constituents of the dried fruit oil were limonene (34.84%), terpinen-4-ol (15.75%), sabinene (11.02%), *α*-Phellandrene (7.10%), cis-*β*-Ocimene (4.74%) and *γ*-Terpinene (4.52%). Drying significantly altered the chemical composition of the essential oil, resulting in marked decreases in the relative abundance of α-phellandrene (19.75% to 7.10%), *β*-myrcene (3.54% to 2.16%), and linalool (2.88% to 1.78%), whereas the relative percentages of limonene (29.52% to 34.84%) and terpinen-4-ol (4.44% to 15.75%) increased substantially. An oil-in-water nanoemulsion containing the essential oil from dried fruits (NE-ZLO) was developed using an optimized mixed-surfactant system with a hydrophilic–lipophilic balance (HLB) of 12.0–15.0 and concentration of 20–26%. Eleven nanoemulsion formulations (NE-ZLO-1 to NE-ZLO-11) were characterized in terms of droplet size, polydispersity index, and zeta potential. NE-ZLO-9 showed the most favorable physicochemical properties while requiring the lowest surfactant concentration. Its stability was evaluated under refrigerated (4 °C), ambient, accelerated (45 °C), and light-exposure conditions compared with that of free essential oil (ZLO). NE-ZLO-9 showed higher apparent limonene-equivalent retention than free ZLO during storage, especially under accelerated and continuous light-exposure conditions. After incorporation into oral care prototypes, the benefits of nanoencapsulation were more pronounced in mouthwash, while the toothpaste matrix itself provided substantial protection against the loss of volatile constituents. These findings suggest that nanoencapsulation and the formulation matrix are important factors in protecting ZLO during storage and support its further development as a natural ingredient in oral care products.

## 1. Introduction

*Z. limonella*, commonly known as Ma-kwaen, is an aromatic plant belonging to the Rutaceae family and is widely distributed throughout Southeast Asia, particularly in northern Thailand [1]. Its fruits possess a characteristic citrus-like aroma and have long been used as both a culinary spice and a traditional remedy for various ailments [2]. In recent years, the essential oil obtained from *Z. limonella* has received considerable attention as a natural ingredient for food, pharmaceutical, and personal care products because of its pleasant aroma and diverse biological activities. Gas chromatography–mass spectrometry (GC–MS) analyses have shown that ZLO consists predominantly of monoterpene hydrocarbons and oxygenated monoterpenes. Limonene is the predominant constituent, followed by sabinene, β-pinene, α-phellandrene, and terpinen-4-ol. However, the relative abundance of these compounds varies depending on the plant part, harvesting conditions, and extraction method [2]. In addition, ZLO exhibits antibacterial activity against a broad spectrum of Gram-positive and Gram-negative bacteria, including multidrug-resistant strains, while the whole essential oil often exhibits greater antibacterial effects than its individual major constituents [3]. Moreover, ZLO also shows potential for oral healthcare applications because of its antimicrobial activity. Limonene, the major constituent of ZLO, has demonstrated inhibitory effects against oral pathogens, including *Streptococcus mutans*, *Streptococcus salivarius*, *Lactobacillus casei*, and *Candida* spp. [4]. In addition, the antioxidant capacity of ZLO has been confirmed by both chemical and cell-based assays, with limonene identified as one of the major contributors to its free radical-scavenging properties [3]. These results suggest that ZLO has high potential as a naturally occurring bioactive ingredient. One of the major limitations of essential oils in oral care formulations is their poor physicochemical stability. They are highly susceptible to volatilization, oxidation, and chemical degradation during storage, particularly when exposed to heat, light, and oxygen, which can reduce both their chemical quality and biological efficacy [5,6]. Nanoencapsulation has therefore emerged as a promising strategy to enhance the aqueous dispersibility of essential oils, protect them from environmental degradation, and enable controlled release. Previous studies have demonstrated that encapsulation approaches including microencapsulation and nanoemulsion systems improve both the stability and antimicrobial activity of essential oils [7,8,9]. Furthermore, nanoemulsion systems have been investigated as delivery systems for essential oils in oral care applications, with potential benefits for their stability, dispersion, and antimicrobial performance [10]. However, limited information is available on the influence of nanoencapsulation on the physicochemical stability and chemical retention of ZLO after its incorporation into oral care formulations such as mouthwash and toothpaste. Therefore, this study was designed with two main objectives. The first was to develop and optimize an oil-in-water nanoemulsion containing ZLO by selecting an appropriate hydrophilic–lipophilic balance (HLB) and surfactant concentration, and to characterize its physicochemical properties. The second was to compare the storage stability of the free ZLO and the nanoemulsion before and after incorporation into toothpaste and mouthwash formulations. Storage stability was evaluated over 12 weeks under refrigerated (4 °C), ambient, accelerated (45 °C), and continuous light-exposure conditions by monitoring physical appearance, physicochemical properties, and chemical retention. Limonene, the predominant volatile constituent of ZLO, was used as a reference compound, and apparent limonene-equivalent retention was determined as a preliminary indicator of chemical retention during storage. The findings provide formulation insights into the use of nanoemulsion systems for improving the storage stability of ZLO in oral care formulations.

## 2. Results

### 2.1. Extraction and Chemical Composition of ZLO

**Figure 1:**
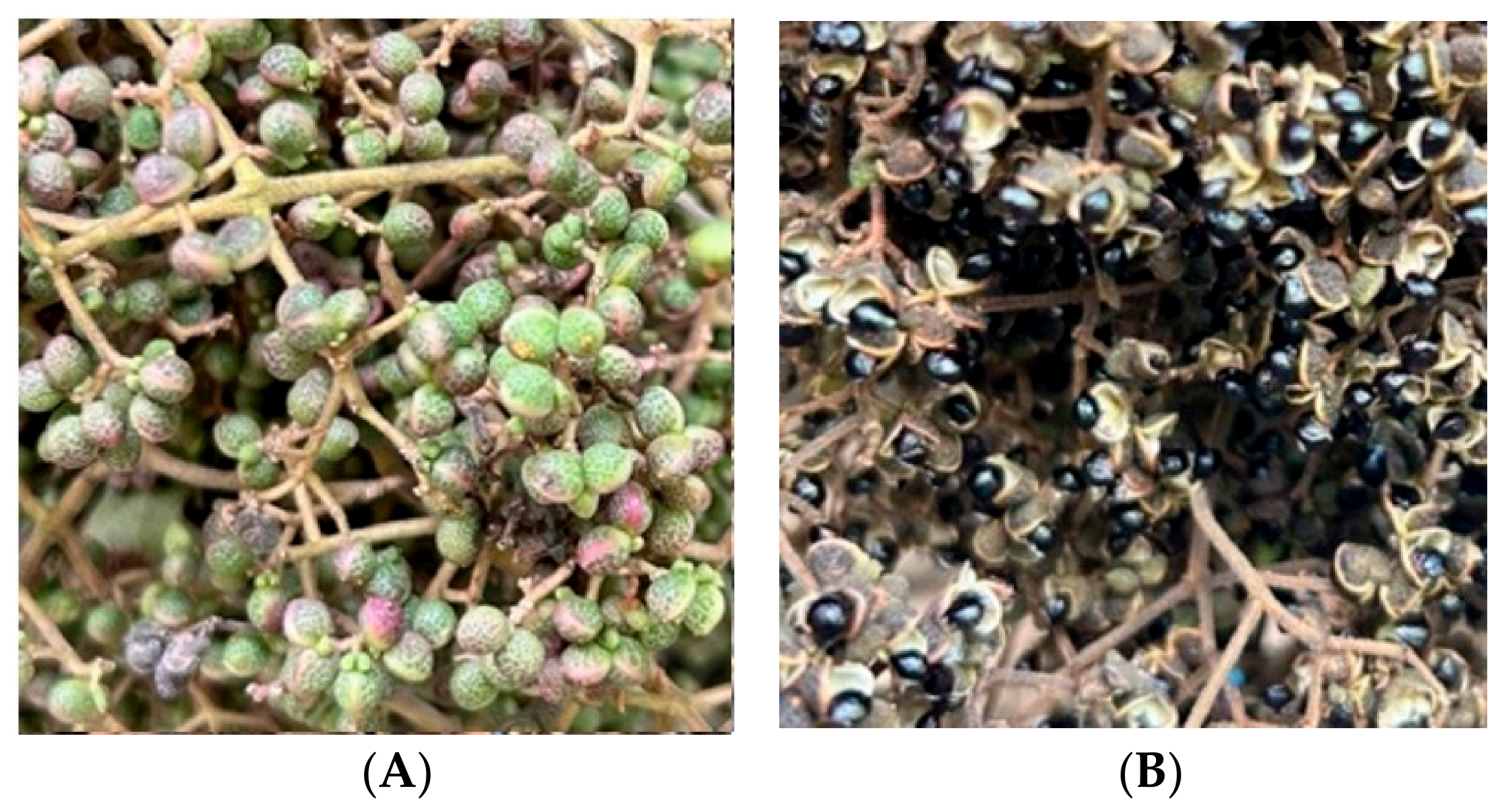
Appearance of fresh (**A**) and shade-dried (**B**) *Z. limonella* fruits.

**Figure 2:**
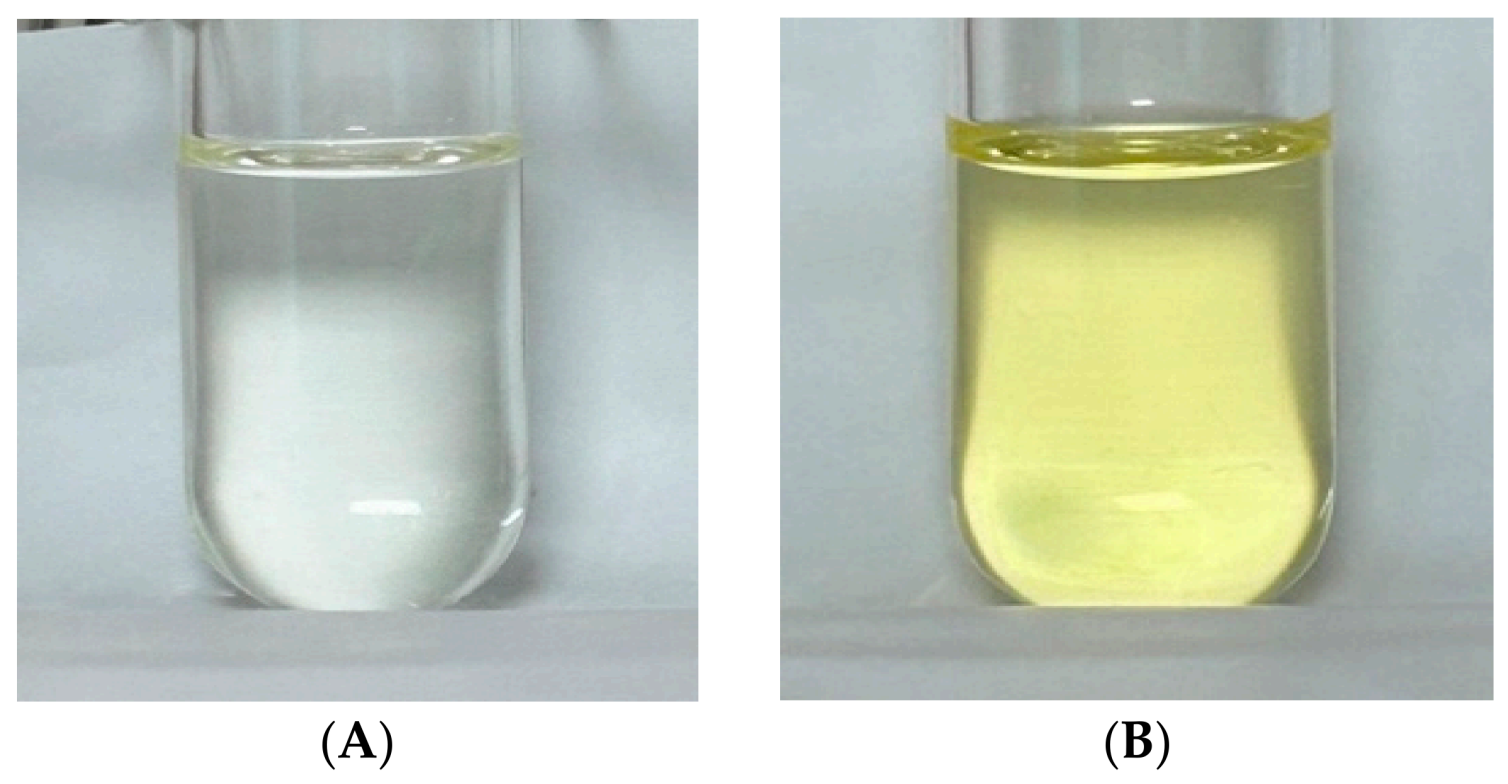
Visual appearance of essential oils extracted from fresh (**A**) and shade-dried (**B**) *Z. limonella* fruits.

The fresh fruits (Figure 1A) had green, intact pericarps and compact fruit clusters. After drying, the fruits (Figure 1B) developed darker brown, fully dehiscent pericarps that exposed the black seeds. The essential oils extracted from fresh and dried *Z. limonella* fruits differed in appearance (Figure 2). The essential oil obtained from fresh fruits was nearly colorless, whereas that extracted from dried fruits was clear pale yellow. These color changes may be associated with chemical alterations induced by the drying process, including the partial oxidation of oxidation-sensitive volatile constituents further supporting that drying promotes the conversion of monoterpene hydrocarbons to oxygenated derivatives. Moreover, drying also affected the essential oil yield, which was higher in dried fruits (10.00%) than in fresh fruits (8.33%). The increased yield may be attributed to the moisture removal, which increases the relative proportion of essential oil in the dry biomass and facilitates hydrodistillation by improving the disruption of secretory tissues and enhancing mass transfer during extraction. Similar results have been reported for several essential oil-producing plants, in which moderate drying enhances essential oil extraction by increasing cell permeability and reducing water disturbance [11]. However, excessive drying may promote thermal oxidation and/or volatilization of thermolabile terpenoids thereby reducing essential oil yield and altering its chemical composition [12].

**Figure 3:**
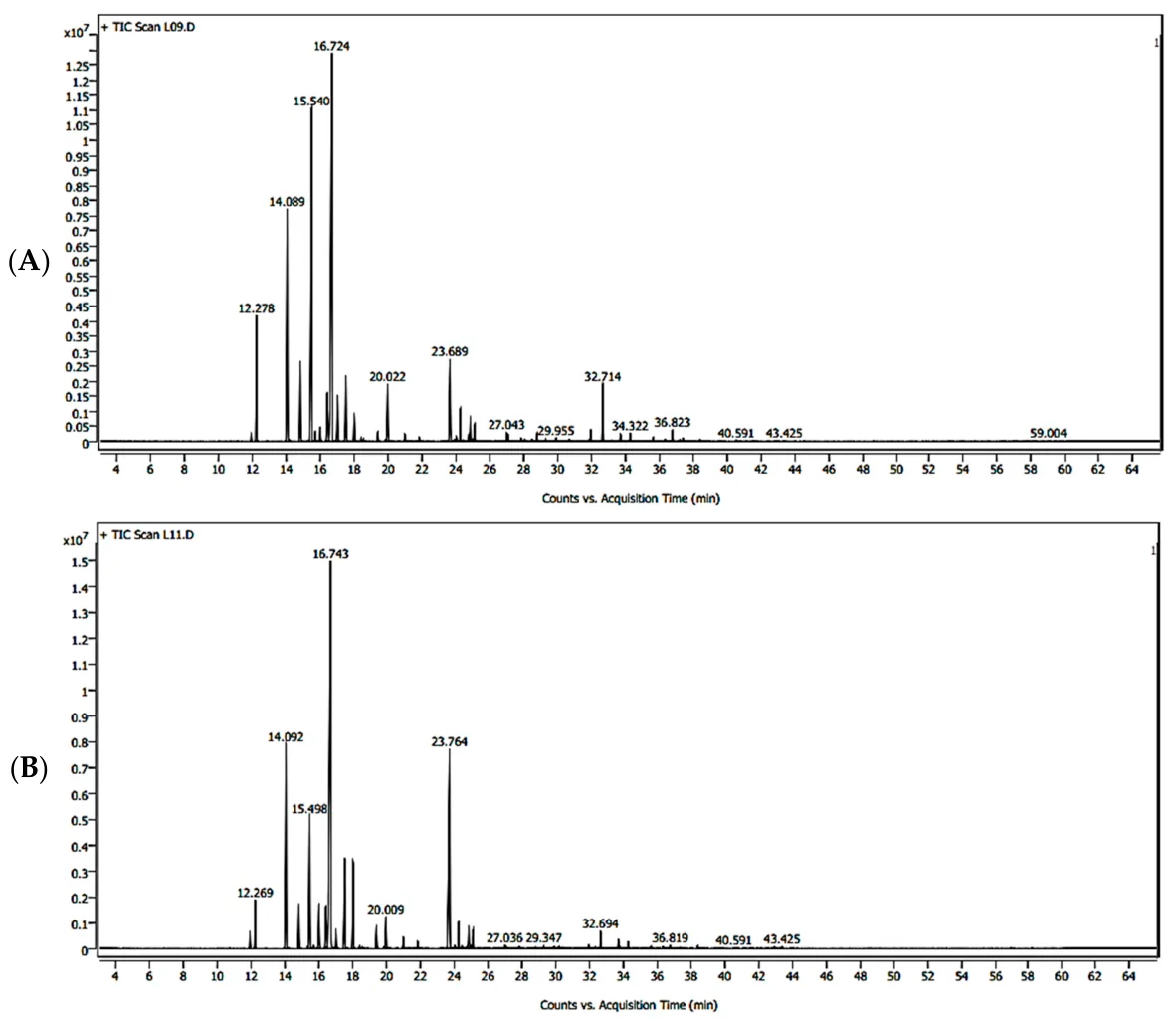
GC–MS chromatograms of ZLO extracted from fresh (**A**) and shade-dried (**B**).

Gas chromatography–mass spectrometry (GC–MS) analysis of ZLO from fresh (ZLO-1) and dried (ZLO-2) *Z. limonella* fruits is presented in Figure 3 and Table 1. The calculated retention indices (RIcal) were in close agreement with the reference values (RIref), supporting the reliable identification of the detected compounds. Both oils were dominated by monoterpenes, primarily, monoterpene hydrocarbons and oxygenated monoterpenes. However, the drying process altered the relative abundance of individual constituents.

The major constituents of the fresh-fruit oil (ZLO-1) were limonene (29.52%), α-phellandrene (19.75%), sabinene (11.33%), α-pinene (5.50%), cis-β-ocimene (5.04%), terpinen-4-ol (4.44%), β-myrcene (3.54%), *p*-cymene (3.05%), linalool (2.88%) and geranyl acetate (2.86%). The volatile profile changed considerably after dehydration. Limonene remained the predominant constituent, increasing to 34.84%, while terpinen-4-ol increased markedly to 15.75%. In addition, moderate increases were observed in γ-terpinene, α-terpinene and terpinolene. In contrast, α-phellandrene decreased markedly from 19.75% to 7.10%, while α-pinene (5.50% to 2.30%), β-myrcene (3.54% to 2.16%), linalool (2.88% to 1.78%), and geranyl acetate (2.86% to 0.91%) also decreased. In comparison, sabinene (11.33% to 11.02%), cis-β-ocimene (5.04% to 4.74%), and *p*-cymene (3.05% to 3.04%) showed relatively small changes. A marked decrease in α-phellandrene, a highly volatile monoterpene hydrocarbon, may be associated with the loss of volatile compounds and/or chemical changes during drying. The reduction in the relative abundance of α-phellandrene following drying is consistent with previous reports on essential oils of *Pituranthos chloranthus* [13] and *Zingiber officinale* [14], supporting the susceptibility of this compound to thermal processing. On the other hand, limonene became relatively more abundant (29.52% to 34.84%) due, most probably, to a relative enrichment effect (the preferential loss of more volatile compounds) rather than an absolute concentration increase. This observation has been recorded for several fragrant species, and drying preferentially removes the most volatile components in a way that alters their relative composition. Additionally, the 3.5-fold increase in terpinen-4-ol (from 4.44% to 15.75%) may be attributed to oxidative and hydrolytic transformations occurring during the drying process. Similar increases in terpinen-4-ol due to drying have been reported for *Melissa officinalis* [15]; these changes may reflect differences in the relative composition of volatile constituents induced by the drying process, including possible volatilization and oxidative changes.

**Figure 4:**
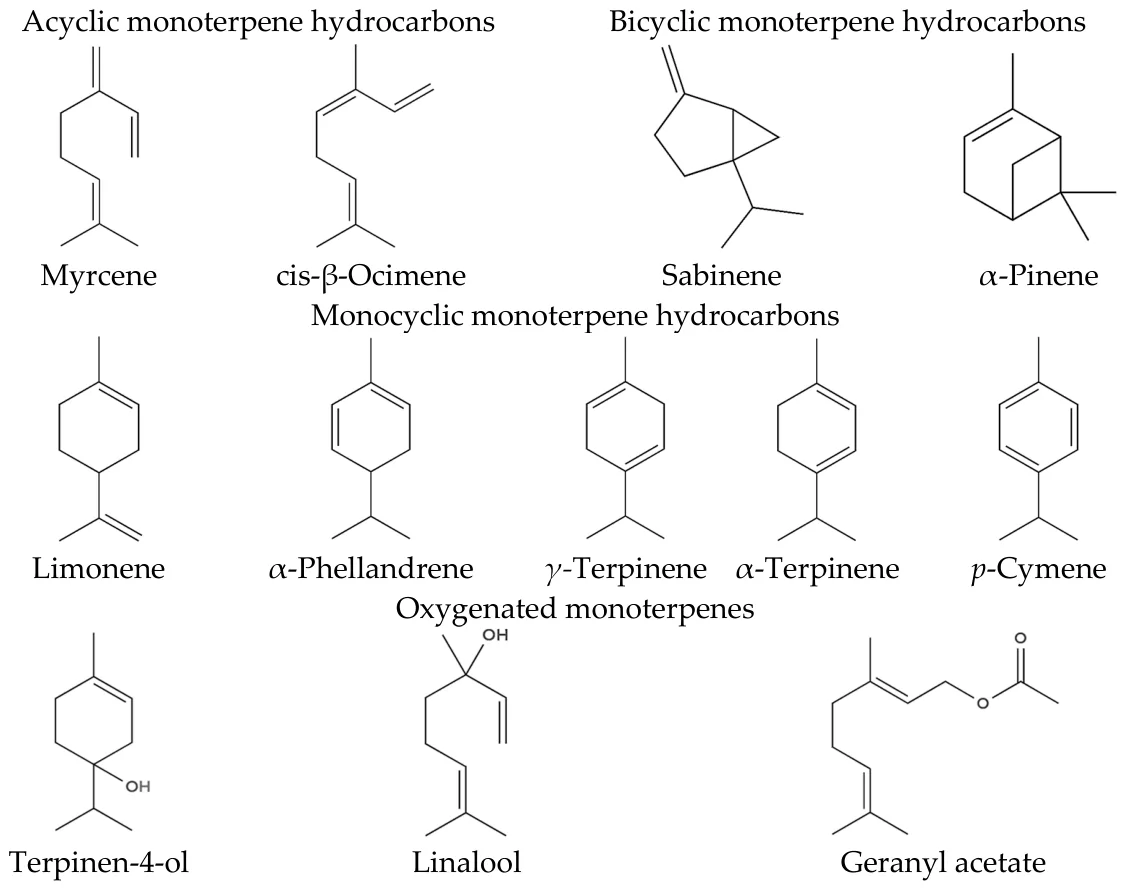
Chemical structures and classification of major volatile constituents identified in ZLO.

These compositional changes are likely to influence the sensory properties and biological activities of ZLO. The essential oil extracted from fresh fruits contained a higher relative abundance of α-phellandrene and other monoterpene hydrocarbons, which are generally associated with fresh, bright and citrus-like aroma. In contrast, the essential oil from dried fruit was enriched in terpinene-4-ol, an oxygenated monoterpene, that is commonly associated with warmer, herbaceous notes. In addition to the modified aroma profile, these compositional changes may also influence the biological properties of ZLO. Monoterpene hydrocarbons (e.g., α-phellandrene and limonene) usually exhibit weak antimicrobial activities, while oxygenated monoterpenes, particularly terpinen-4-ol, have been reported to yield stronger antibacterial, anti-inflammatory, and antioxidant activities due to their higher polarity [16]. Thus, from these results, the dried fruit oil (ZLO-2) was advanced for further development. In addition to their favorable chemical composition, particularly the higher terpinen-4-ol content, dried fruits also provide significant practical advantages for large-scale production. Drying naturally increases the shelf life of raw materials by decreasing water content thus preventing microbial growth. It also enables processing during off seasons, provides better consistency from batch to batch and simplifies mechanical comminution and hydrodistillation which, in turn, increases overall process efficiency supporting lower cost [17]. As a result, ZLO-2 was selected as the source oil for the preparation of NE-ZLOs and subsequent incorporation into oral care formulations. The chemical structures and classification of the major volatile constituents identified in ZLO are shown in Figure 4.

### 2.2. Nanoemulsion Preparation and Characterization

#### 2.2.1. Solubility Screening of ZLO in Carrier Oils

Selecting an appropriate carrier oil is a critical step in nanoemulsion formulation because it directly influences the solubilization, droplet formation, encapsulation efficiency, and long-term stability of essential oils [18]. Carrier oils with a greater capacity to dissolve lipophilic bioactive compounds form more stable nanoemulsions with reduce phase separation during storage [18,19]. As previous reports by Abdalla et al. [20] and Li et al. [21] reported that limonene (the main component of ZLO), exhibits greater solubility in jojoba oil. Particularly, jojoba oil differs from other triglyceride oils in that it is primarily composed of long-chain liquid wax esters [22], giving it enhanced oxidative stability and thermal resistance [23]. This characteristic structure not only assures strong affinity with lipophilic terpenoids but also protects limonene and other oxidation-sensitive monoterpenes from degradation, favoring improving physicochemical stability of the nanoemulsion. Similar results were reported by Kubitschek-KM and Zero [24], who successfully used jojoba oil as the carrier oil for essential oil nanoemulsions. Based on these findings, jojoba oil was selected for subsequent nanoemulsion formulation and hydrophilic–lipophilic balance (HLB) optimization.

#### 2.2.2. Effect of Surfactant Hydrophilic–Lipophilic Balance (HLB) on Nanoemulsion Properties

**Figure 5:**
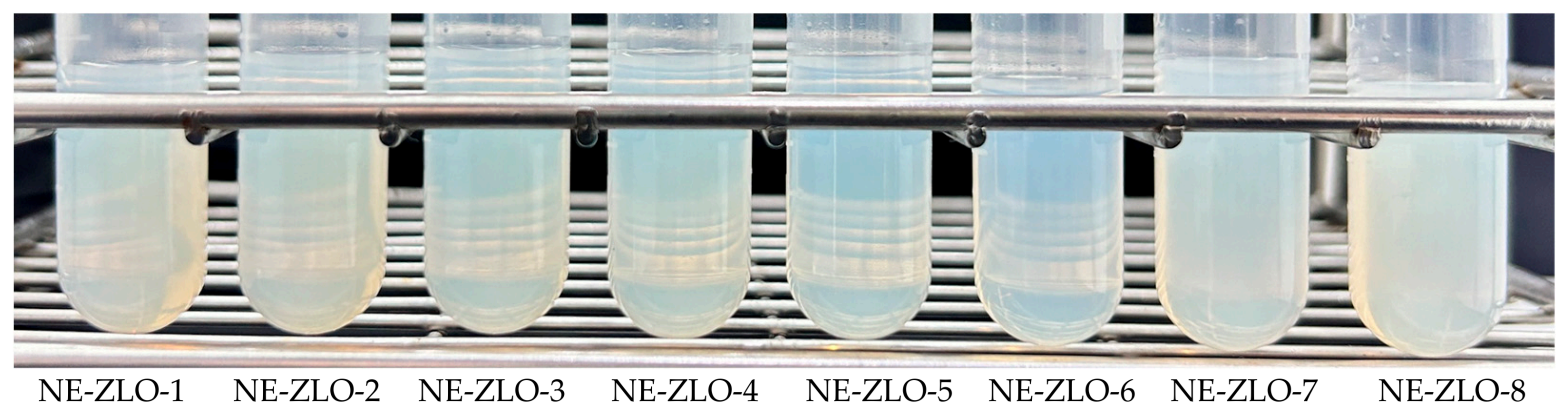
Visual appearance of NE-ZLOs prepared using surfactant systems with different HLB values.

The HLB of surfactant systems is one of the most important parameters for nanoemulsion formulation, as it affects the affinity of surfactants for both the oil and aqueous phases, interfacial tension, droplet formation, and emulsion stabilization. Therefore, determining the required HLB (rHLB) is crucial for choosing an appropriate surfactant blend that yields nanodroplets with small sizes, narrow size distributions, and acceptable physical stability. In this study, three structurally different nonionic surfactants, PEG-40 hydrogenated castor oil (PEG-40 HCO), Tween 80, and Span 80, were selected to allow adjustment of the overall HLB of the surfactant system while providing complementary interfacial functions. PEG-40 HCO, with its bulky polyoxyethylene head group and hydrogenated castor oil hydrophobic moiety, enhances the solubilization of lipophilic compounds and improves the loading capacity of essential oils. Tween 80, a highly hydrophilic surfactant, effectively reduces interfacial tension and facilitates droplet formation, whereas Span 80, a lipophilic surfactant with a low HLB value, improves surfactant packing at the oil–water interface. Furthermore, these surfactants are expected to form a compact and flexible interfacial film, thereby improving emulsion stability. Based on a previous report [24], the rHLB of jojoba oil is 10–11; therefore, emulsifier blends with HLB values ranging from 10.0 to 15.0 were prepared to identify the optimum HLB for nanoemulsion formulation. Based on visual observation, all formulations prepared with different surfactant HLB values appeared generally similar, as homogeneous translucent dispersion without phase separation (Figure 5). Among the formulations, NE-ZLO-3 to NE-ZLO-7 (HLB = 12.0–14.5) appeared more translucent, whereas NE-ZLO-1, NE-ZLO-2, and NE-ZLO-8 appeared opaque. The higher transparency of NE-ZLO-4 to NE-ZLO-6 suggests the formation of finer droplets, which scatter less visible light than larger droplets. Therefore, particle size, polydispersity index (PDI), and zeta potential were further evaluated to determine the optimum HLB value and confirm the physical stability of the formulations. Figure 5 shows the effect of the HLB value on the physicochemical properties of nanoemulsions (NE-ZLOs). As the HLB value increased from NE-ZLO-1 to NE-ZLO-6, the droplet sizes were gradually reduced from 62.57 ± 0.98 nm to 47.37 ± 0.49 nm, with significant differences among formulations (*p* < 0.05) (Table 2). This tendency suggests that surfactant systems with high HLB values resulted in more efficient reduction of the interfacial tension between the oil and aqueous phases, droplet disruption during emulsification, and generation of smaller (nano-sized) droplets. Conversely, a further increase in the HLB led to an expansion in droplet size, with values increasing up to 54.20 ± 0.17 nm and 64.77 ± 0.25 nm for NE-ZLO-7 and NE-ZLO-8, respectively. All formulations differed significantly in droplet size except NE-ZLO-1 and NE-ZLO-2 (*p* < 0.05). This suggests that too high hydrophilic surfactant blend reduced its stabilizing the oil–water interface, causing droplet enlargement and reduced emulsification efficiency.

The polydispersity index (PDI) is a measure of the width of particle size distribution and is widely used to assess the uniformity of colloidal systems. Higher PDI values indicate a broader particle size distribution and consequently, greater heterogeneity in droplet size, whereas lower PDI values indicate a narrower distribution and a more homogeneous (monodisperse) droplet population. In general, PDI values greater than 0.5 indicate highly polydisperse systems [25]. In the current study, the PDI values ranged from 0.174 ± 0.00 to 0.322 ± 0.01, with significant differences among formulations (*p* < 0.05)**.** Low PDI values (0.174–0.180) were observed for formulations NE-ZLO-5 and NE-ZLO-6, suggesting a narrow droplet size distribution. On the other hand, NE-ZLO-8 exhibited the highest PDI (0.322 ± 0.01), indicating a broader size distribution and greater heterogeneity. NE-ZLO-6 had a significantly lower PDI than NE-ZLO-8 (*p* < 0.05), whereas the remaining formulations showed comparable PDI values. These results indicate that the HLB value producing the smallest droplets also generated a more homogeneous nanoemulsion system. It is suggested that at HLB 14.0, surfactant molecules packed more efficiently at the oil–water interface, leading to simultaneous reductions in droplet size and PDI.

Zeta potential is the electrical potential at the surface surrounding dispersed droplets, which is widely accepted as an indicator of colloidal stability. Higher absolute zeta potential values refer to stronger electrostatic repulsion between droplets, inhibits aggregation and coalescence. In general, a zeta potential value of +/− 30 mV or greater is sufficient to provide good electrostatic stabilization of a dispersed system [26]. In the present study, the zeta potential values of the nanoemulsions ranged from −22.10 ± 0.87 to −36.03 ± 0.64 mV and differed significantly among formulations (*p* < 0.05). The magnitude of the negative surface charge generally increased with increasing HLB value. The absolute zeta potential values of formulations NE-ZLO-7 and NE-ZLO-8 were the highest (−34.93 and −36.03 mV, respectively), which may enhance resistance to droplet aggregation. However, despite their higher absolute zeta potential values, these formulations exhibited significantly larger droplet sizes and higher PDI than NE-ZLO-6 (*p* < 0.05). This finding suggested that zeta potential alone was insufficient to predict nanoemulsion performance. Instead, interfacial stabilization balance, surfactant adsorption efficiency, and compatibility of the surfactant blend with the essential oil phase seemed to be more influential on droplet size and overall formulation stability.

#### 2.2.3. Effect of Surfactant Concentration on Nanoemulsion Properties

**Figure 6:**
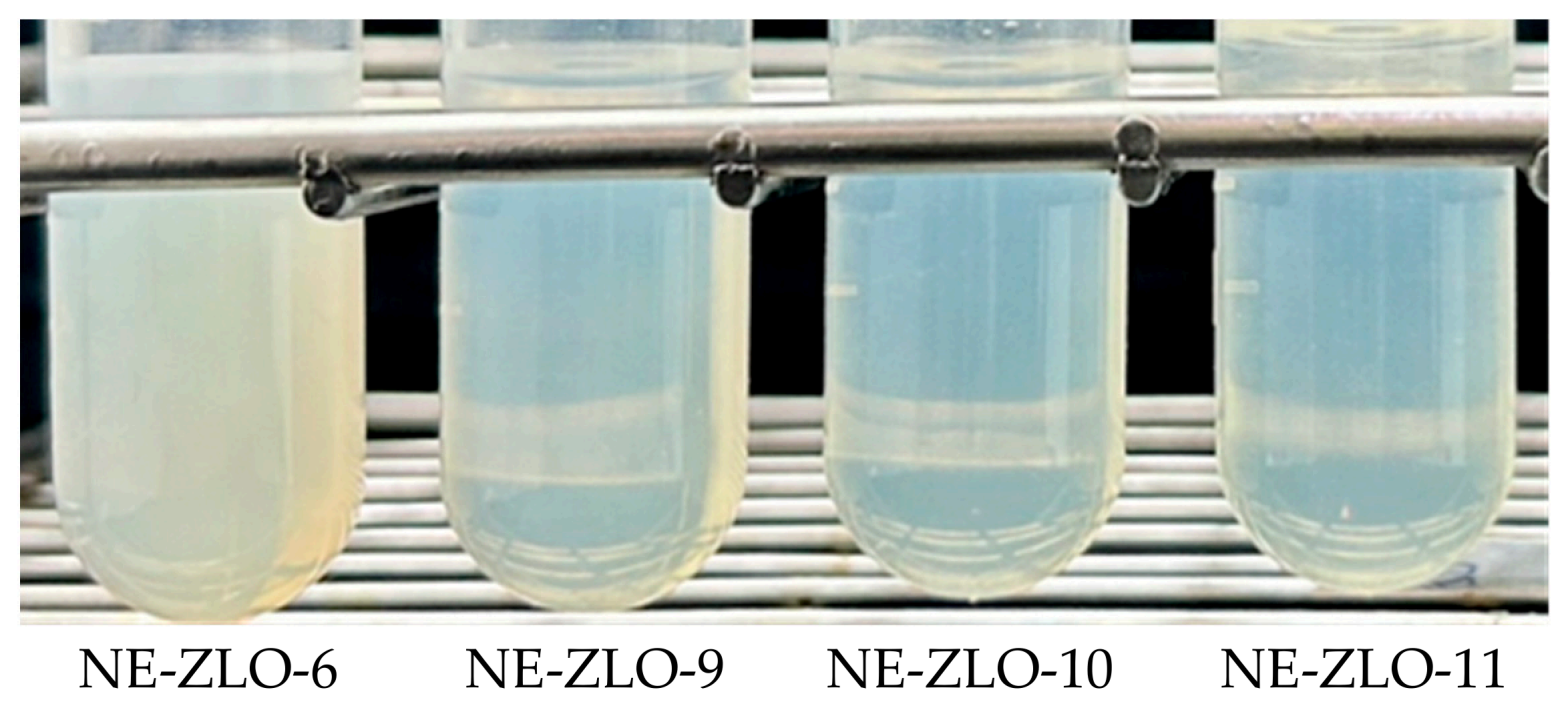
Visual appearance of NE-ZLOs prepared with different surfactant concentrations.

The surfactant concentration is critical parameter in nanoemulsion formulation because a sufficient amount of surfactant is required to reduce interfacial tension between the oil and aqueous phases and to stabilize formed droplets during emulsification. In this study, formulation NE-ZLO-6 had 20% surfactant, whereas formulations NE-ZLO-9-11 contained progressively higher surfactant concentrations of 22%, 24%, and 26%, respectively. These results are presented in Table 3 and Figure 6, from which it can be seen that the size of nanoemulsions decreased with increasing surfactant concentration. The differences in droplet size among formulations were statistically significant (*p* < 0.05). The minimum mean droplet size was decreased from 47.37 ± 0.49 nm in NE-ZLO-6 to 40.83 ± 0.06 nm, and gradually passed through the values in NE-ZLO-9 (39.53 ± 0.15 nm), NE-ZLO-10 (36.23 ± 0.32 nm) which also followed a distinct downward trend of droplet size with surfactant concentration as previously reported by Chuacharoen et al. [27] and Sarheed et al. [28]. The smaller droplet size can be attributed to the greater interfacial coverage provided by surfactants as well as preventing coalescence of droplets during homogenization due to more surfactant molecules adsorbed at the oil-water interface. All formulations differed significantly in particle size (*p* < 0.05). A significant reduction in particle size marked by over 14% for droplet sizes was achieved with 20% (NE-ZLO-6) to 22% (NE-ZLO-9) surfactant concentrations. At higher concentrations of surfactant, the droplet sizes changed slowly and gradually. The implication was that saturation of the interfacial surfactant concentration had likely been reached.

The PDI values for all nanoemulsion formulations depicted in this study (0.180 ± 0.02 to 0.225 ± 0.01) are less than the threshold of ≤0.3 indicating that the systems are relatively homogenous. Although PDI differed significantly among formulations (*p* < 0.05), formulation NE-ZLO-6 exhibited the narrowest size distribution (0.180 ± 0.02). The PDI values increased slightly with increasing surfactant concentrations. However, all formulations exhibited PDI values within the acceptable range for nanoemulsions, indicating that the increase in surfactant concentration had only a minor effect on droplet size distribution and did not compromise formulation homogeneity.

The zeta potential of the nanoemulsions ranged from −29.70 ± 0.44 to −32.50 ± 0.89 mV. Although a significant difference in zeta potential was detected among formulations (*p* < 0.05), increasing in surfactant concentration from 20% (NE-ZLO-6) to 26% (NE-ZLO-11) had only a minor effect on the zeta potential. The most negative zeta potential was observed for NE-ZLO-10 (−32.50 ± 0.89 mV) and other formulations exhibit values close to −30 mV. Only NE-ZLO-10 and NE-ZLO-11 differed significantly (*p* < 0.05), while the remaining formulations were statistically comparable. These results suggest that surfactant concentration had little influence on the surface charge of the nanoemulsion droplets. Since Tween 80, PEG-40 hydrogenated castor oil, and Span 80 are nonionic surfactants, the stability of the nanoemulsions is attributed primarily to steric stabilization provided by the hydrated surfactant layer surrounding the droplets rather than electrostatic repulsion [26].

Overall, a surfactant concentration of 22% provided sufficient interfacial coverage, as further increases to 24% and 26% resulted in only marginal improvements in the physicochemical properties of the nanoemulsions. Moreover, the use of a lower surfactant concentration is generally preferred in cosmetic formulations because it may reduce the risk of surfactant-induced skin or mucosal irritation. Accordingly, NE-ZLO-9 was selected for subsequent stability studies, as it produced the smallest droplet size (40.83 ± 0.06 nm) with an acceptable PDI (0.207 ± 0.01) using only 22% surfactant.

### 2.3. Stability During Storage

Free ZLO and NE-ZLO-9 were evaluated for their storage stability under four different conditions; refrigeration (4 °C), room temperature in the laboratory light (RT), accelerated storage at 45 °C, and a continuous exposure to light for 12 weeks. Physical stability was assessed by visual appearance for both samples, while the nanoemulsion was further characterized by particle size, PDI, and zeta potential measurements. To assess chemical stability, the retention of major volatile compound, limonene, in free essential oil and nanoemulsion during storage was compared before and after incorporation into toothpaste and mouthwash final formulations under the same storage conditions.

#### 2.3.1. Physical Stability

**Figure 7:**
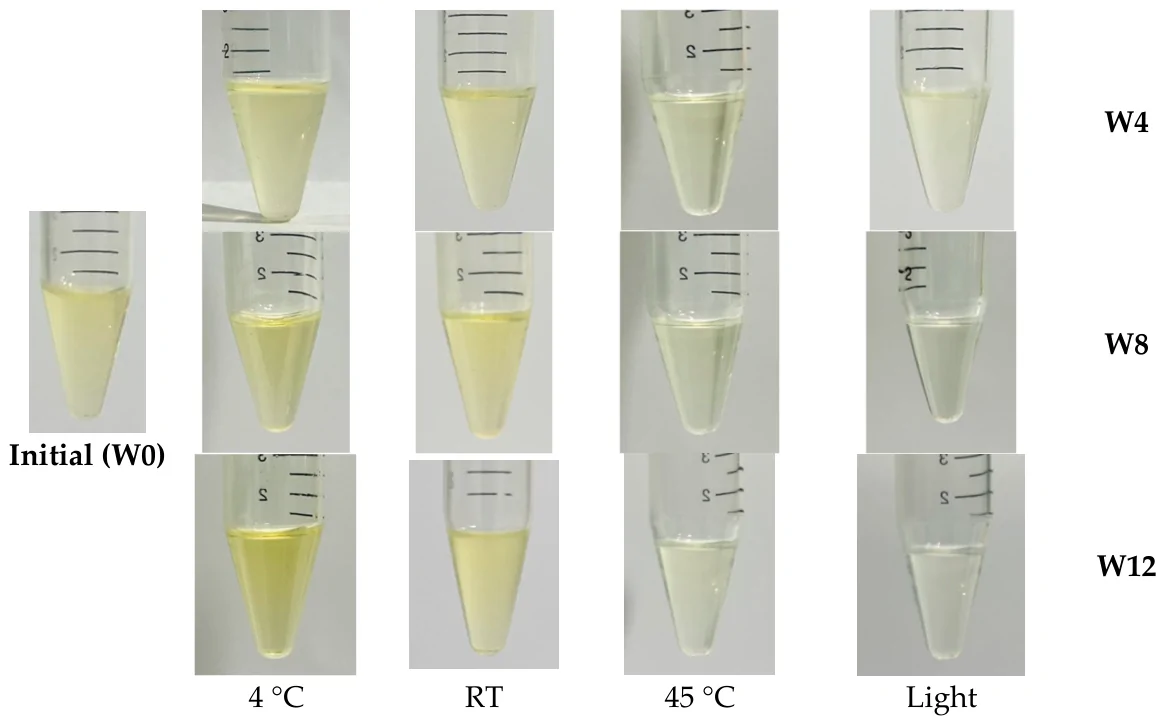
Visual appearance of free ZLO during storage under different conditions (4 °C, Room Temperature, 45 °C, and light exposure) for 12 weeks.

**Figure 8:**
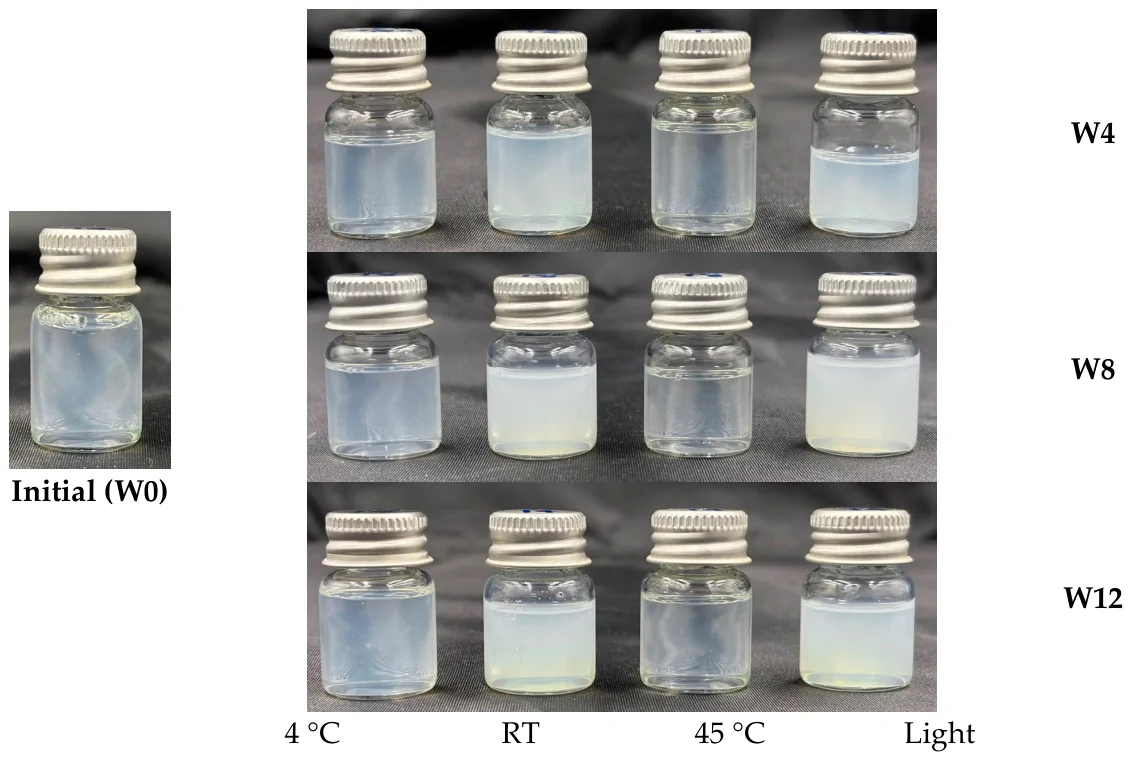
Visual appearance of NE-ZLO-9 during storage under different conditions (4 °C, Room Temperature (RT), 45 °C, and light exposure) for 12 weeks.

During storage, noticeable differences in the appearance of free ZLO and NE-ZLO-9 were observed by visual inspection. The characteristic pale-yellow color of free ZLO gradually faded, especially upon exposure to both elevated temperatures and continuous light, where the discoloration was noted as early as 2 weeks and progressed to an almost completely colorless solution by week 12 (Figure 7). The fading is likely associated with thermal- and photo-oxidative degradation of unprotected ZLO, leading to the oxidation of naturally occurring chromophoric compounds. While NE-ZLO-9 retained its original appearance after storage without phase separation, only a slight increase in turbidity was observed, and the nanoemulsion remained homogeneously dispersed without visible oil separation, creaming, sedimentation, or precipitation (Figure 8). The increase in turbidity observed for NE-ZLO-9 under stress conditions may be attributed to changes in the interfacial structure of the nanoemulsion. The absence of discoloration in NE-ZLO-9 indicates that the encapsulation system helped preserve its visual stability under the tested storage conditions.

The initial pH of NE-ZLO-9 was 6.10 ± 0.01 and decreased to 5.96 ± 0.02, 5.92 ± 0.01, 5.60 ± 0.01, and 5.81 ± 0.02 after 12 weeks of storage at 4 °C, RT, 45 °C, and under continuous light exposure, respectively, as shown in Table 4. The smallest change was observed at 4 °C (ΔpH = −0.14), whereas the greatest decrease occurred at 45 °C (ΔpH = −0.50), indicating that elevated temperature had the greatest effect on pH stability. This reduction in pH may reflect chemical changes in the essential oil or other ingredients within the formulation.

These visual observations were supported by the physicochemical data presented in Table 5, Table 6 and Table 7. The initial particle size, PDI, and zeta potential of NE-ZLO-9 were 40.83 nm, 0.211, and −31.10 mV, respectively. Among all storage conditions, 4 °C exhibited the greatest physical stability, as evidenced by relatively constant particle size (40.83–37.50 nm), a slight decrease in PDI (0.211–0.186), and negligible changes in zeta potential (−31.10 to −30.67 mV). Within refrigerated storage, particle size decreased significantly during storage (*p* < 0.05), whereas zeta potential remained unchanged (*p* > 0.05).

At room temperature under ambient laboratory light, the particle size gradually increased to 79.27 nm after 12 weeks, with significant increases after Week 4 (*p* < 0.05), whereas zeta potential showed no significant changes throughout storage (*p* > 0.05), suggesting gradual droplet growth over time.

Interestingly, the formulation stored at 45 °C exhibited a smaller particle size (32.17 nm) and lower PDI (0.105), indicating a more homogeneous droplet population. However, prolonged storage significantly reduced the absolute zeta potential to −23.77 mV (*p* < 0.05), suggesting that the interfacial stability of NE-ZLO-9 gradually decreased under thermal exposure.

The greatest increase in particle size and PDI was observed under continuous light exposure, where particle size and PDI increased significantly to 99.13 nm and 0.343, respectively (*p* < 0.05), whereas zeta potential changed only slightly to −29.63 mV (*p* > 0.05). The greater increase in particle size under continuous light, compared with room-temperature storage under ambient light and storage at 45 °C in the dark, suggests that continuous light had a greater impact on nanoemulsion stability than heat alone. At Week 12, the particle size under continuous light was significantly larger than that under the other storage conditions (*p* < 0.05). In contrast, storage at 45 °C caused only minor changes in droplet size, indicating that the interfacial film remained largely intact under thermal stress alone.

Refrigerated storage (4 °C) provided the greatest physical stability, with no significant changes in zeta potential and only minor variations in particle size and PDI throughout the 12-week storage period (*p* > 0.05). These results suggest that low-temperature storage effectively preserved the physicochemical characteristics of the nanoemulsion. The stability may be attributed to decreased molecular mobility and increased viscosity of the continuous phase at low temperature, which limit droplet collision, coalescence, and oxidation. This temperature-dependent behavior has also been reported for nanoemulsions containing *Lavandula dentata* or *Myristica fragrans* essential oils, in which storage temperature and duration significantly influenced the physicochemical properties and chemical stability of the nanoemulsions, with elevated temperature accelerating destabilization and the loss of volatile constituents [29]. Previous reports have shown that prolonged light exposure can alter the interfacial properties of essential oil nanoemulsions, promoting droplet aggregation and changes in turbidity; for example, in citrus essential oil nanoemulsions, exposure to light, heat, and oxygen has been reported to compromise physicochemical stability, with changes in droplet interfacial characteristics promoting destabilization processes such as coalescence and Ostwald ripening [30]. Consistent with this mechanism, continuous light exposure produced significantly greater increases in particle size and PDI than the other storage conditions (*p* < 0.05). Storage at 45 °C resulted in a gradual decrease in particle size and PDI throughout storage, although greater chemical degradation observed under this condition. Similar behavior has occasionally been reported for nanoemulsions, where the low viscosity of the continuous phase combined with increased molecular mobility at elevated temperatures may facilitate droplet reorganization resulting in a slight reduction in particle size distribution despite the faster oxidation of volatile compounds [31,32]. In contrast, a significant reduction in zeta potential was observed only under storage at 45 °C, decreasing from −31.10 to −23.77 mV (*p* < 0.05), whereas no significant changes were observed under refrigerated, room-temperature, or continuous light storage. This reduction reflects a gradual loss of electrostatic repulsion between droplets. The changes in particle size and zeta potential were not always consistent which illustrated that particle size alone cannot fully predict long-term colloidal stability. Thus, it is important to assess nanoemulsion stability using several complementary parameters such as the visual appearance, particle size, PDI and zeta potential [26].

#### 2.3.2. Chemical Stability of ZLO in Different Formulations

**Figure 9:**
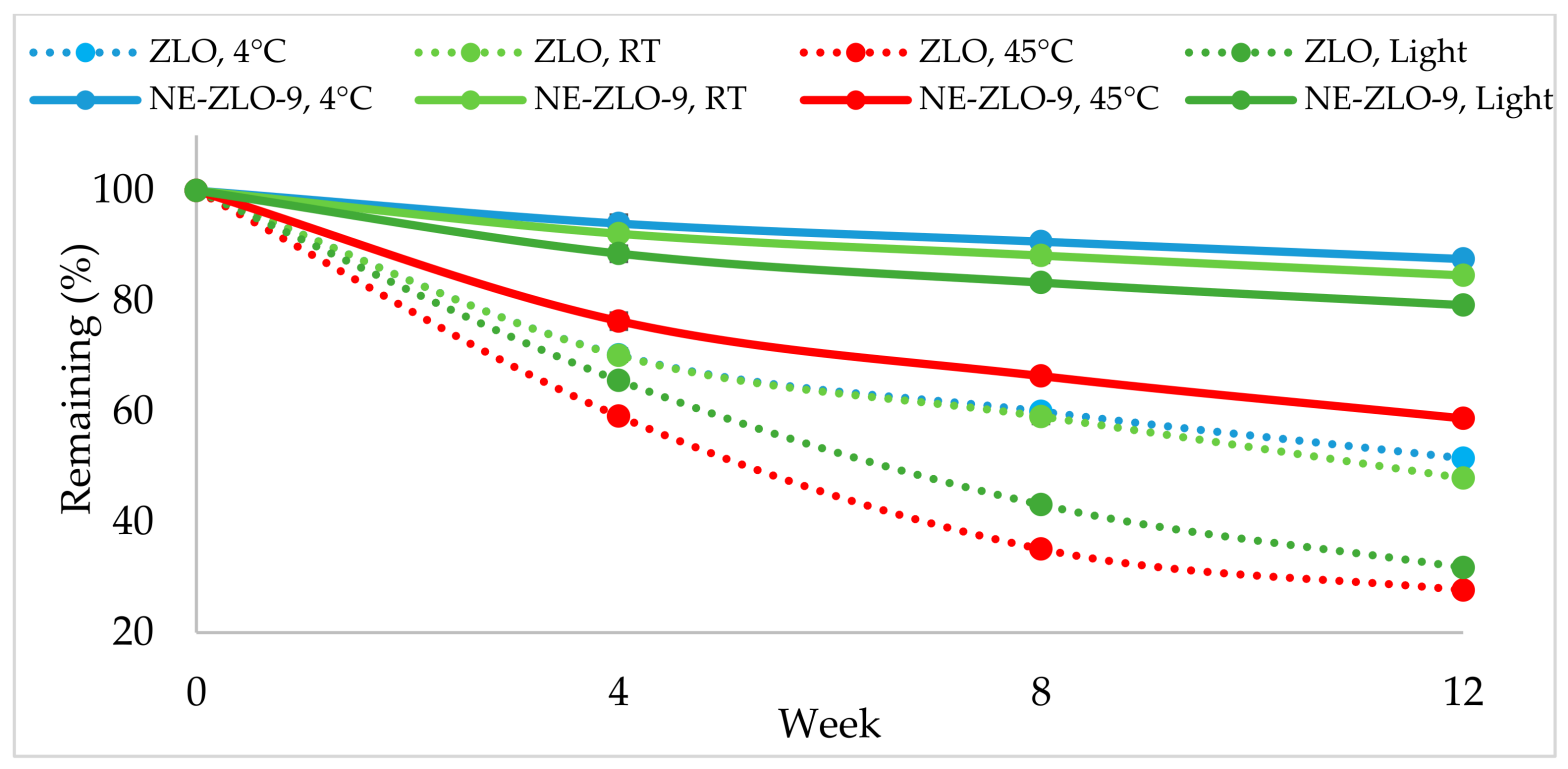
Time-dependent Limonene-equivalent retention in free ZLO and NE-ZLO-9 during storage under different conditions.

During storage, free ZLO gradually lost its characteristic pale-yellow color (Figure 7). Particularly, under accelerated storage conditions (45 °C) and continuous light exposure, discoloration became evident within the first 4 weeks and progressed until week 12. The loss of the characteristic yellow color coincided with a significant decrease in limonene equivalent retention, which was attributed to the continuous loss of volatile compounds caused by volatilization and subsequent oxidative degradation [33,34]. Chemical stability testing showed that free ZLO retained only 49.71% and 46.30% of limonene after 12 weeks under refrigerated and room-temperature storage, respectively, while retention dropped sharply to 21.98% at 45 °C and 28.99% under continuous light exposure (Table 8 and Figure 9). It should be emphasized that the apparent limonene-equivalent retention reported herein was based on absorbance at 252 nm and represents an empirical optical stability index of the formulation rather than a definitive quantification of intact limonene or its degradation kinetics. These results show that heat and light significantly accelerate the degradation of volatile terpenoids through a combination of volatilization, autoxidation, and photooxidation.

In contrast, NE-ZLO-9 exhibited significantly higher apparent limonene-equivalent retention than free ZLO under all storage conditions (*p* < 0.05). After 12 weeks, retention remained as high as 87.65% at refrigerated storage and 85.06% at room temperature, while 58.84% and 78.94% of limonene were still preserved under 45 °C and continuous light exposure, respectively. Compared with free ZLO, nanoencapsulation enhanced retention by approximately 1.8-fold under refrigerated and room temperature storage, 2.7-fold under accelerated storage, and 2.7-fold under continuous light exposure, demonstrating a strong protective effect under environmental stress. The enhanced stability of NE-ZLO-9 can be attributed to encapsulation of the essential oil within surfactant-stabilized nano-sized droplets. The interfacial surfactant layer may reduce direct exposure of the encapsulated oil to environmental factors and limit the loss of volatile constituents [35].

Overall, the stability of NE-ZLO-9 depended on the storage condition and the physicochemical parameter evaluated. Refrigerated storage (4 °C) best preserved both the physicochemical properties and visual appearance of the nanoemulsion, whereas continuous light exposure primarily promoted colloidal destabilization. In contrast, elevated temperature had a greater impact on chemical retention than on physical stability. These findings are consistent with previous studies demonstrating that nanoencapsulation improves the stability of essential oils by minimizing droplet coalescence and protecting volatile compounds against oxidation and volatilization under environmental stress [26,29].

**Figure 10:**
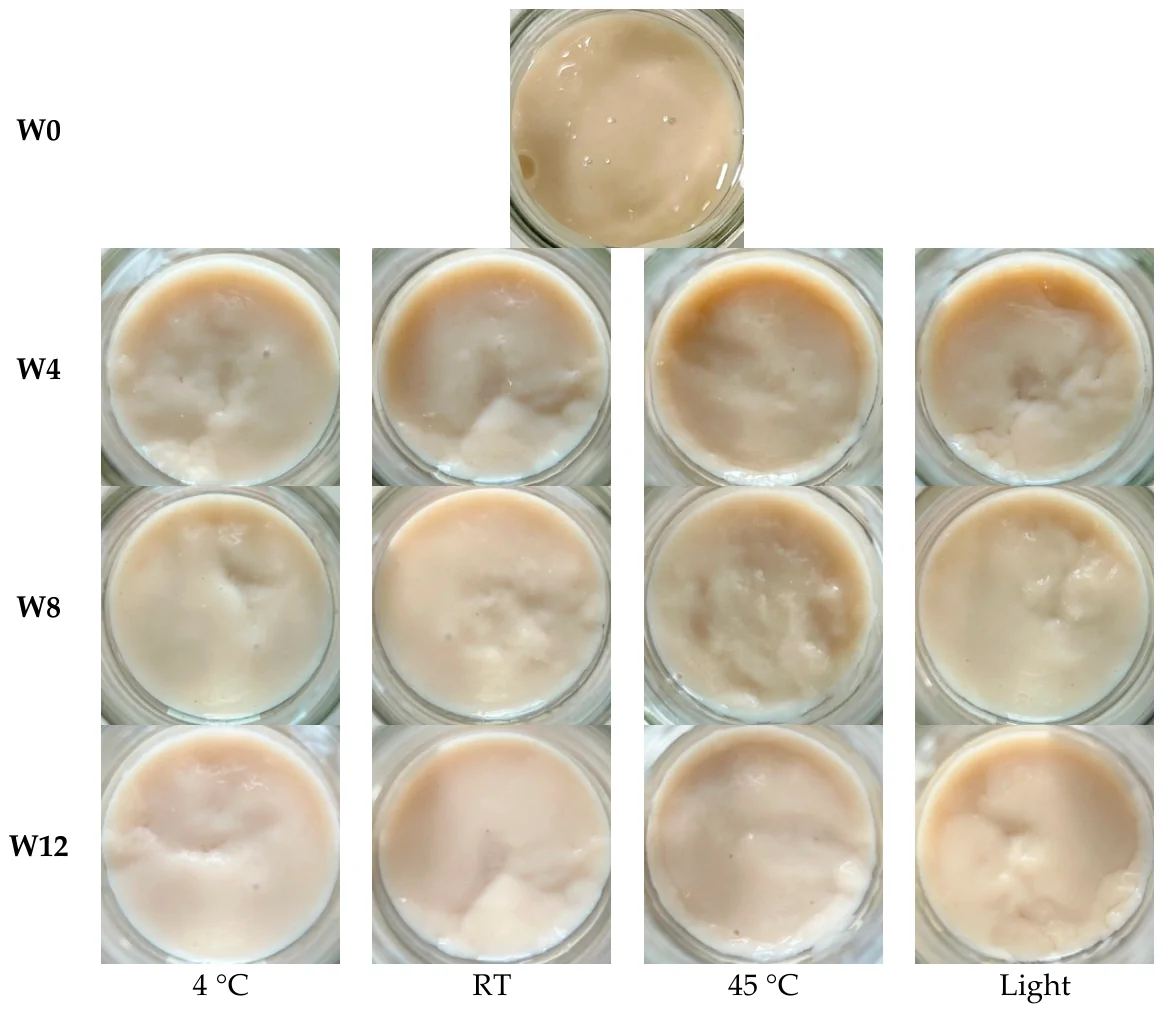
Visual appearance of toothpaste formulations containing free ZLO during storage under different conditions.

**Figure 11:**
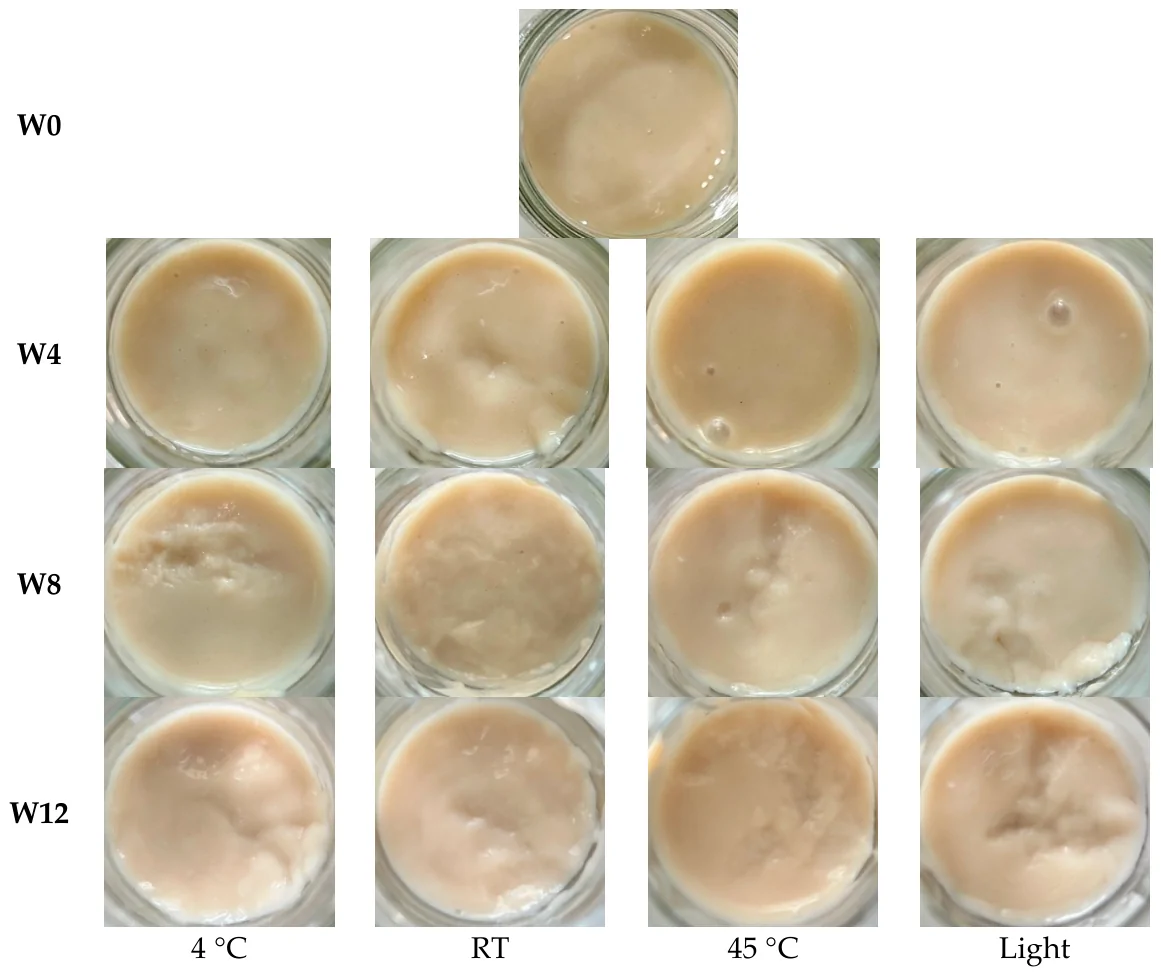
Visual appearance of toothpaste formulations containing NE-ZLO-9 during storage under different conditions.

**Figure 12:**
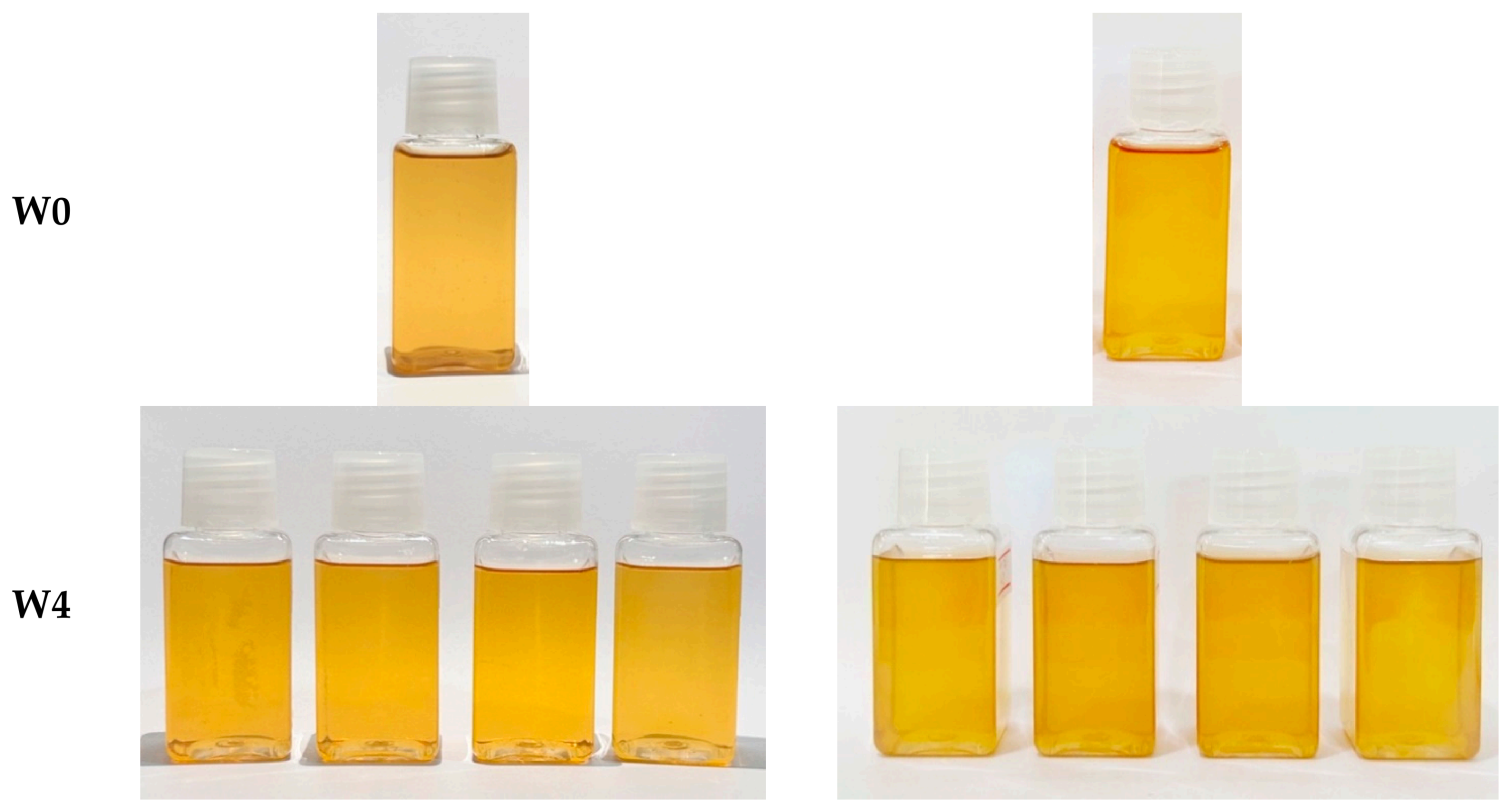

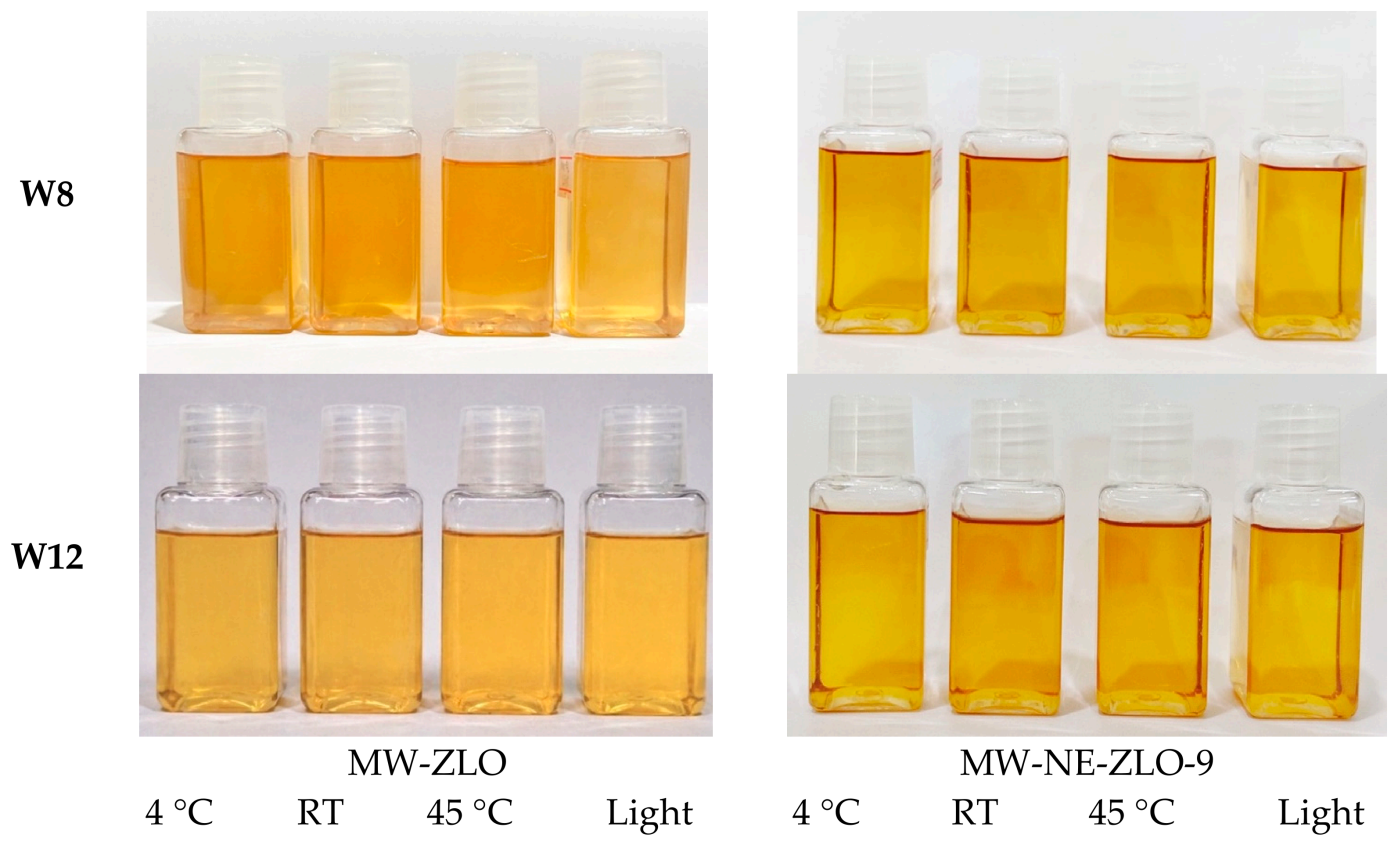
Visual appearance of mouthwash formulations containing free ZLO and NE-ZLO-9 during storage under different conditions.

The stability after incorporation of free ZLO and NE-ZLO-9 into toothpaste and mouthwash formulation matrixes was further evaluated. Toothpaste and mouthwash preparations had different physical properties. The prepared toothpaste and mouthwash showed distinct physical characteristics. The toothpaste was a homogeneous, smooth, beige-colored cream appearance without visible phase separation (Figure 10 and Figure 11). On the other hand, the mouthwash was a clear, transparent, yellow solution with no visible signs of precipitation (Figure 12). The initial viscosities of TP-ZLO and TP-NE-ZLO-9 were 35.91 ± 0.01 and 34.84 ± 0.01 Pa s, respectively (Table 9). On the other hand, it was impossible to accurately measure the viscosity of MW-ZLO and MW-NE-ZLO-9 because both formulations were free-flowing liquids whose viscosity fell below the measurable range of the rotational viscometer configuration used (S21 spindle, 1 rpm). Therefore, a capillary viscometer would be more appropriate for obtaining reliable viscosity measurements of these low-viscosity mouthwash formulations. The differences in consistency between these products may influence their stability patterns. Storage conditions had an effect on the viscosity of toothpastes (Table 9). At the end of the study period, only the toothpastes stored at 45 °C showed a large increase in viscosity, which was probably caused by changes in the toothpaste matrix.

The pH of the toothpaste and mouthwash formulations was evaluated over the 12-week study to assess physicochemical stability. The initial pH was 6.90 for TP-ZLO and 6.65 for TP-NE-ZLO-9, while MW-ZLO and MW-NE-ZLO-9 had pH values of 6.85 and 6.79, respectively. The pH levels in both formulations gradually changed over the course of the 12-week storage period (Table 10 and Table 11). However, throughout the study, despite the decreases in pH in both the toothpaste and mouthwash formulations, the pH of each formulation remained above 5.5, a level commonly associated with an increased risk of enamel demineralization when pH falls below it. The largest changes typically occurred at higher temperatures and under continuous light exposure. In particular, the pH levels in the mouthwash formulations showed greater decreases, especially under continuous light exposure. Overall, the data indicate that both temperature and light exposure affected the pH of both types of formulations.

**Figure 13:**
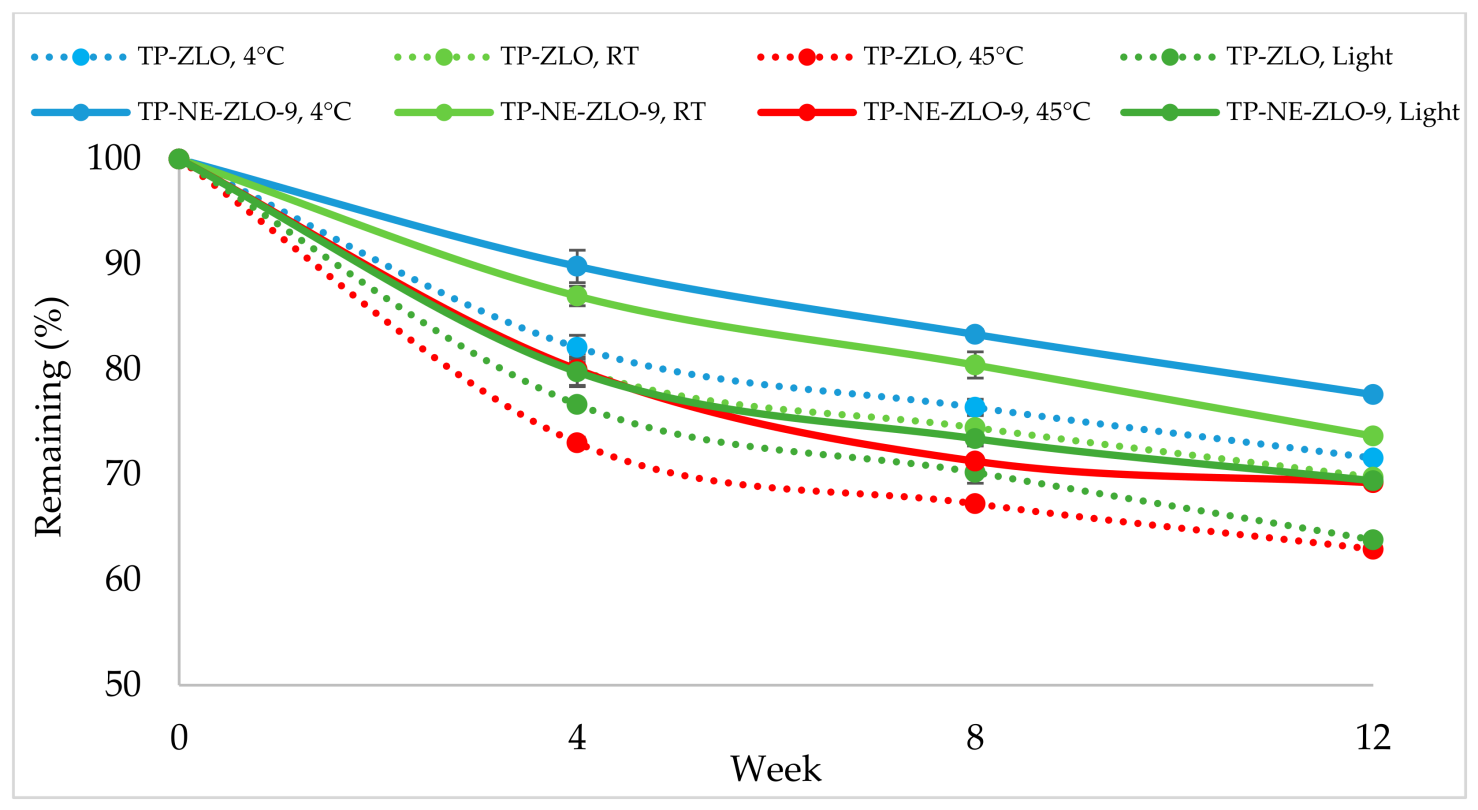
Time-dependent Limonene-equivalent retention in toothpaste formulations containing free ZLO and NE-ZLO-9 during storage.

Toothpaste (TP) exhibited the greatest intrinsic stability, with over 60% limonene-equivalent retention under all storage conditions. At week 12, TP-ZLO showed 62.92–71.59% retention, whereas TP-NE-ZLO-9 showed 69.24–77.62% retention, indicating relatively small differences between the two systems (Table 12 and Figure 13). The increased stability of both toothpaste formulations is likely related to the viscosity of the semi-solid matrix, which may provide a protective environment. Diffusion-reaction models of emulsion oxidation indicate that increasing viscosity may decrease molecular mobility and oxygen transport through the aqueous domain [36], while the emulsifier layer may provide an additional mass-transfer barrier to oxygen transport into the oil phase [37]. However, oxygen diffusion was not directly measured in the present study; therefore, these mechanisms should be considered possible contributing factors rather than direct evidence of improved stability. The relatively small difference in retention between TP-ZLO and TP-NE-ZLO-9 indicates that the toothpaste matrix itself plays an important role in stabilizing ZLO and may limit the additional benefit of nanoencapsulation.

**Figure 14:**
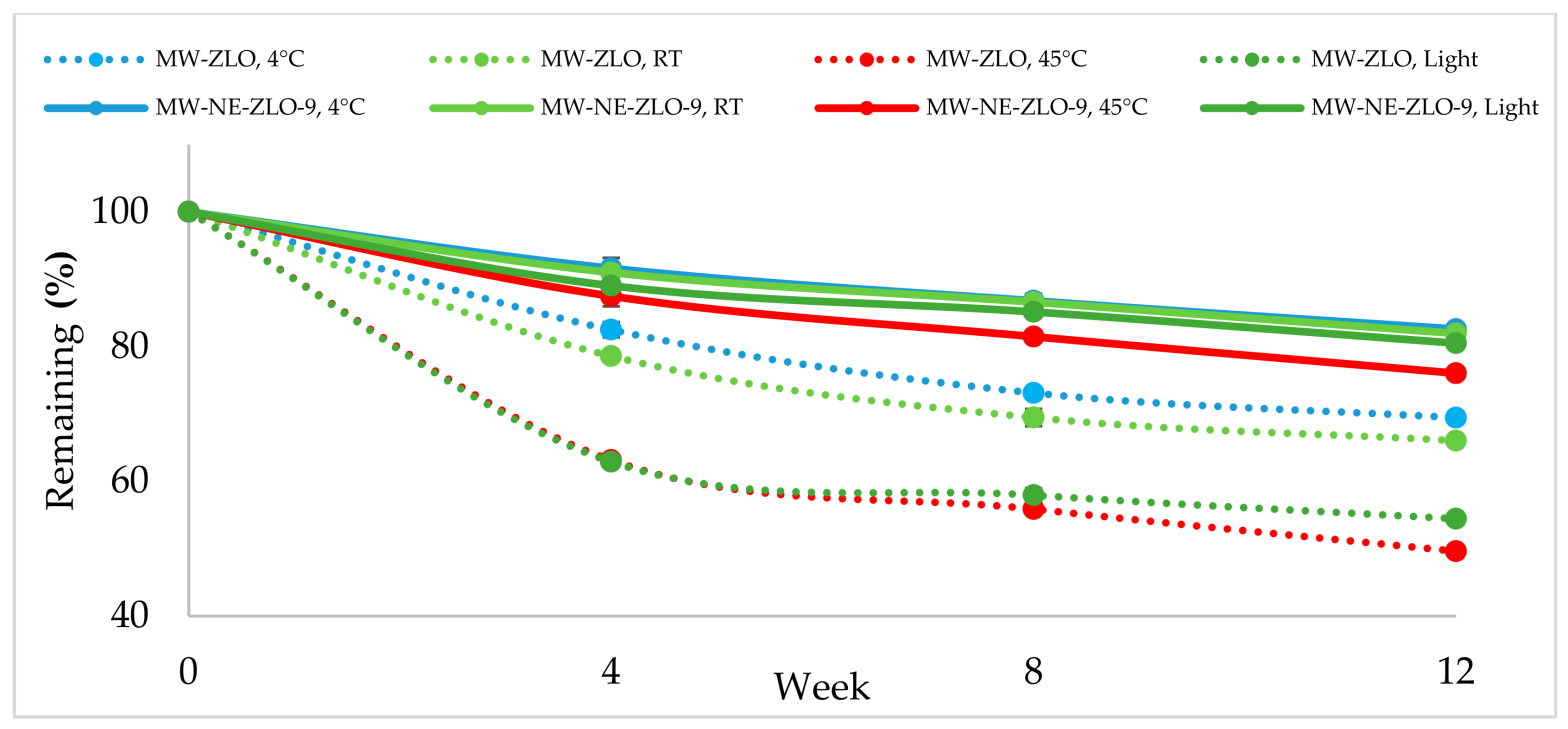
Time-dependent Limonene-equivalent retention in mouthwash formulations containing free ZLO and NE-ZLO-9 during storage.

In contrast, the mouthwash (MW) formulation showed a greater benefit from nanoencapsulation. As a free-flowing aqueous formulation, the mouthwash provided a different physicochemical environment from the viscous semi-solid toothpaste. Consequently, MW-NE-ZLO-9 consistently showed higher limonene-equivalent retention than MW-ZLO throughout storage. At the end of the study, retention ranged from 67.72–77.44% for MW-NE-ZLO-9 compared with 62.82–71.78% for MW-ZLO, with the largest differences observed under accelerated temperature and continuous light exposure (Table 13 and Figure 14). These findings suggest that nanoemulsification provided a more apparent additional protective effect in the mouthwash formulation than in the toothpaste formulation.

Overall, nanoencapsulation and the formulation matrix appear to provide complementary stabilization effects. Nanoencapsulation improved the retention of ZLO constituents, while the formulation matrix provided additional protection during storage. Among the formulations investigated, toothpaste provided the greatest inherent protection, whereas the additional benefit of nanoencapsulation was more apparent in the mouthwash formulation.

### 2.4. Antimicrobial Activity

The antibacterial activity of ZLO and NE-ZLO-9 against *S. mutans* was evaluated using agar well diffusion and broth microdilution assays, followed by scanning electron microscopy (SEM) to examine treatment-induced morphological changes. However, the absence of an inhibition zone does not always imply a lack of antibacterial activity. This may be because agar diffusion depends on both the release and distribution of antimicrobial components through the agar. It is possible that incorporation of ZLO into the nanoemulsion reduced the immediate availability of volatile constituents during the assay. The broth microdilution results confirmed that NE-ZLO-9 retained antibacterial activity, with a minimum inhibitory concentration (MIC) of 3.125%, although its minimum bactericidal concentration (MBC) was not reached within the tested concentration range (>50%) (Table 14). Free ZLO showed a lower MIC (0.078%) and an MBC of 0.3125%. The lower apparent activity of NE-ZLO-9 may be associated with decreased initial availability of compounds within the nanoemulsion matrix. In the finished prototypes, NE-ZLO-9 was used at 1.0% (*w*/*w*), equivalent to approximately 0.10% (*w*/*w*) ZLO. This level is below the MIC of NE-ZLO-9 (3.125%), so the antimicrobial activity of the nanoemulsion should not be assumed for the finished toothpaste or mouthwash, which were not directly tested. Although the free-ZLO level (0.10% *w*/*w*) is close to the MIC of raw ZLO (0.078% *v*/*v*), direct comparison is limited by the different concentration units and formulation matrices.

**Figure 15:**
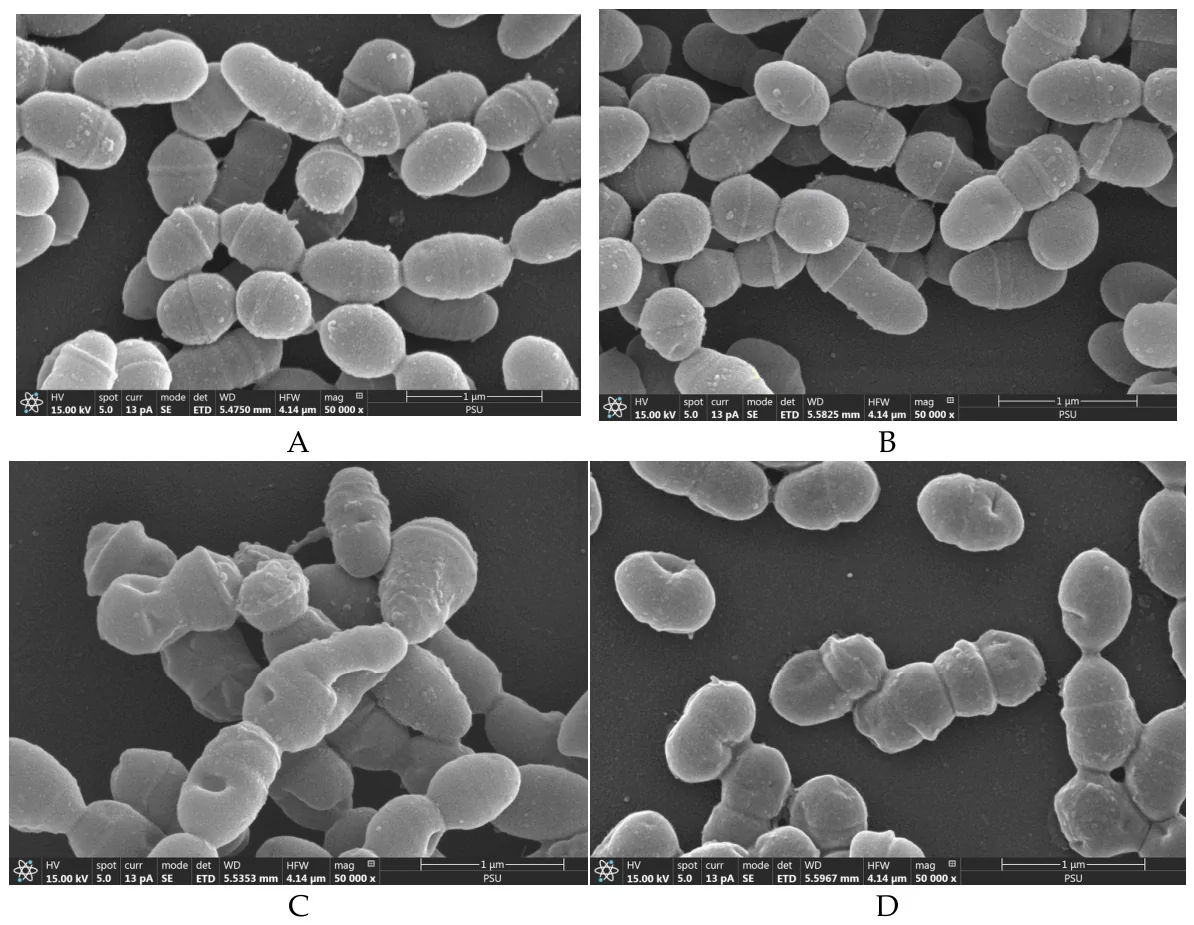
Scanning electron microscopy (SEM) images of *S. mutans* following treatment with (**A**) untreated control, (**B**) DMSO, (**C**) ZLO, and (**D**) NE-ZLO-9. Images were obtained at ×50,000 magnification.

The effects of ZLO and NE-ZLO-9 on the morphology of *S. mutans* were further examined using SEM (Figure 15). Untreated cells showed relatively uniform morphology with smooth surfaces. Cells treated with DMSO showed a generally similar morphology, although some surface irregularities were observed. Cells exposed to NE-ZLO-9 displayed some surface irregularities and morphological changes, however most cells continued to maintain their original form. In contrast, cells treated with ZLO exhibited significant morphological alterations such as uneven cell wall surfaces, indentations and deformations. These observations are consistent with the antibacterial results, in which ZLO showed greater antibacterial activity than NE-ZLO-9 under the tested conditions.

## 3. Materials and Methods

### 3.1. Materials

PEG-40 hydrogenated castor oil (PEG-40 HCO), polysorbate 80 (Tween 80), sorbitan monooleate (Span 80), xanthan gum, and calcium carbonate were purchased from Chanjao Longevity Co., Ltd. (Bangkok, Thailand). Jojoba oil, glycerin, hydrated silica, hydroxyapatite, titanium dioxide, and propylene glycol were obtained from Namsiang Company Limited (Bangkok, Thailand). Sodium benzoate and sodium chloride were purchased from Chemecosmetics Co., Ltd. (Bangkok, Thailand). Sodium carboxymethyl cellulose was obtained from Krungthepchemi Company Limited (Bangkok, Thailand). Sodium laureth sulfate (Texapon^®^ N8000) was purchased from Sept Chemical Co., Ltd. (Chonburi, Thailand). *n*-Hexane (HPLC grade) was purchased from Qrec (Auckland, New Zealand). *n*-Alkane standards (C8–C25) were obtained from Merck KGaA (Darmstadt, Germany). Limonene standard (purity ≥95%) was purchased from Sigma-Aldrich (Saint Louis, MO, USA), and ethanol was obtained from RCI Labscan Limited (Bangkok, Thailand)**.** Helium gas (99.999% purity) was supplied by Linde Public Company Limited (Bangkok, Thailand). Deionized water was used throughout the experiments.

### 3.2. Plant Materials and Drying Conditions

Fruits of *Zanthoxylum limonella* were collected in November 2025 from Chiang Rai Province, Thailand. The plant material was authenticated, and a voucher specimen (No. CBIS-ZL-001) was deposited in the reference collection of the Cosmetic and Beauty Innovations for Sustainable Development (CBIS), School of Cosmetic Science, Mae Fah Luang University, Thailand. The fruits were divided into two groups to examine the effect of drying on essential oil composition and biological activity. Fresh fruits (ZLO-1) were processed immediately after collection, while the second group (ZLO-2) was shade-dried in a well-ventilated area at ambient temperature for 18 days.

### 3.3. Essential Oil Extraction

Essential oils were extracted separately from fresh and shade-dried *Z. limonella* fruits by hydrodistillation using a 200 L commercial distillation unit. Briefly, 10 kg of fruit was mixed with 80 L of distilled water and hydrodistilled for 8 h. The collected essential oil was dried over anhydrous sodium sulfate to remove residual moisture, filtered, and stored in amber glass vials at 4 °C until further analysis.

The essential oil yield was calculated on a dry weight basis using Equation (1):

Yield (%) = (Weight of essential oil/Weight of plant material) × 100(1)

### 3.4. GC–MS Analysis

The chemical characterization of ZLO was performed by gas chromatography–mass spectrometry (GC–MS) analysis using an Agilent 7890B gas chromatograph coupled with a mass selective detector Agilent 5977A (Agilent Technologies, Santa Clara, CA, USA). A HP-5MS capillary column (30 m × 0.25 mm i.d., 0.25 μm film thickness) was used to achieve separation. The carrier gas was helium at constant flow rate (1.0 mL/min). The injector was kept at 250 °C and the split ratio was 1:50. The oven was held at 60 °C for 2 min, programmed to 250 °C at a rate of 3 °C/min and that temperature was maintained for the last ten minutes. Data were obtained over a scan range of *m*/*z* 30–300. The identification of volatile compounds was carried out using mass spectra obtained from these samples by comparing for the spectra with those in the National Institute of Standards and Technology (NIST) library, and additionally calculated retention indices (RI_cal_) were compared to published retention index data. The method was not validated for the analysis of intact thermally labile peroxide compounds. Retention indices were determined based on a homologous series of *n*-alkanes (C_8_–C_25_) measured under the same chromatographic conditions, using the Van den Dool and Kratz method [38]. The RI_cal_ were determined with the following equation:

RI_cal_ = 100[n + (t_R_(x) − t_R_(n))/(t_R_(n + 1) − t_R_(n))] (2)

where t_R_(x) is the retention time of the target compound, t_R_(n) and t_R_(n + 1) are the retention times of *n*-alkanes eluting immediately before and after this compound (n = carbon number of preceding *n*-alkanes).

Relative abundance of each individual compound was calculated by (peak area/total chromatographic peak area) × 100% using peak area normalization without correction factors.

### 3.5. Preparation of Nanoemulsion

Nanoemulsions loaded with ZLO were prepared using a low-energy emulsification method. The formulation was optimized in two sequential steps. The first step was to determine the appropriate HLB value by preparing formulations with HLB values ranging from 10.0 to 15.0 with a certain amount of emulsifier and the total emulsifier concentration was fixed at 20% (*w*/*w*). The desired HLB values were obtained by adjusting the proportions of Tween 80, Span 80, and PEG-40 hydrogenated castor oil according to the blending rule (Table 15). Following identification of the optimal HLB value, optimization was carried out for total emulsifier concentration (*w*/*w*) by increasing the amount of emulsifier from 20% to 26%, with all preparations retaining the optimal HLB value (Table 15). In all formulations, the concentrations of ZLO, jojoba oil, and glycerin were fixed at 10% (*w*/*w*). For nanoemulsion preparation, the oil phase, consisting of ZLO, jojoba oil, and the surfactant mixture, was heated to 50 °C. The aqueous phase containing glycerin and deionized water was heated separately to the same temperature. The oil phase was then added gradually to the aqueous phase under continuous magnetic stirring at 700 rpm until a homogeneous translucent nanoemulsion was obtained. The prepared nanoemulsions were allowed to equilibrate at room temperature for 24 h before physicochemical characterization.

### 3.6. Characterization of Nanoemulsion

The average droplet size (Z-average), polydispersity index (PDI), and zeta potential of the nanoemulsions were determined by dynamic light scattering (DLS) using a Nano Particle Analyzer SZ-100Z2 (HORIBA Ltd., Kyoto, Japan). Before analysis, samples were diluted 100-fold with distilled water to minimize multiple scattering. Droplet size and PDI were measured using disposable polystyrene cuvettes, whereas zeta potential was determined using a folded capillary cell. All measurements were performed in triplicate at 25 °C after an equilibration period of 2 min, and the results are expressed as the mean ± standard deviation (SD).

### 3.7. Preparation of Toothpaste and Mouthwash

Prototype toothpaste and mouthwash formulations were prepared to examine the stability of free ZLO and NE-ZLO-9 in oral care products. The compositions of the formulations are shown in Table 16 and Table 17, respectively. NE-ZLO-9 contained 10% (*w*/*w*) ZLO; therefore, adding 1.00% (*w*/*w*) NE-ZLO-9 gave a final ZLO concentration of 0.10% (*w*/*w*), which was the same concentration used in the free ZLO formulations.

To prepare the toothpaste, sodium carboxymethyl cellulose and xanthan gum were dispersed in the mixture of deionized water, glycerin and propylene glycol and stirred until a uniform gel base was formed. Sodium benzoate and sodium laureth sulfate (27%) were then added, followed by hydrated silica, calcium carbonate, hydroxyapatite, and titanium dioxide. For TP-ZLO, free ZLO was first mixed with Tween 80 and PEG-40 hydrogenated castor oil and then added to the toothpaste base. For TP-NE-ZLO-9, the nanoemulsion was added directly to the base. Both formulations were gently stirred until smooth and homogeneous.

To prepare the mouthwash, sodium benzoate and sodium chloride were dissolved in deionized water, followed by glycerin. For MW-ZLO, free ZLO was first mixed with Tween 80 and PEG-40 hydrogenated castor oil before gradually adding to the aqueous phase. Then, formulations were stirred until clear and homogeneous.

The prepared products were transferred into airtight containers and allowed to equilibrate at room temperature for 24 h before the storage stability study. The pH of prepared products was measured by using a pH meter (HI2211 HANNA Basic pH/ORP Benchtop Meter, Hanna Instruments, Bangkok, Thailand).

The viscosity of the toothpaste formulations was measured using a Brookfield RVDV-II + PRO viscometer (RVDV2T Extra, Brookfield, Middleboro, MA, USA) equipped with an S21 spindle. Each sample (8.0 mL) was placed in the sample chamber and measured at 1 rpm, corresponding to a shear rate of approximately 1.3 s^−1^, after an equilibration time of 45 s. Measurements were performed in triplicate, and the results were expressed as mean ± SD (cP). The same measurement conditions were initially applied to the mouthwash formulations. However, both MW-ZLO and MW-NE-ZLO-9 were highly fluid and produced torque values below the reliable detection range of the instrument; therefore, their viscosities could not be accurately quantified using this setup. A capillary viscometer, such as an Ostwald or Ubbelohde viscometer, would be more suitable for precise viscosity measurement of these low-viscosity formulations and should be considered in future studies.

### 3.8. Accelerated Storage Stability Test

The stability of free essential oil (ZLO) and its nanoemulsion (NE-ZLO-9) in solution form and after incorporation into oral care formulations (mouthwash and toothpaste) was evaluated over a 12-week under four storage conditions: refrigerated (4 ± 1 °C), ambient (25 ± 2 °C under normal laboratory lighting), accelerated (45 ± 1 °C), and continuous light exposure (approximately 1000 lux at 25 ± 2 °C). Physicochemical and chemical properties were evaluated at weeks 0, 4, 8, and 12. Changes in physical appearance (e.g., discoloration, turbidity, precipitation, creaming and phase separation) were evaluated through visual inspection. Moreover, for nanoemulsion samples, the droplet size, polydispersity index (PDI) and zeta potential of NE-ZLO-9 were determined by dynamic light scattering (DLS) using Nano Particle Analyzer SZ-100Z2 (HORIBA Ltd., Kyoto, Japan). The samples were diluted 100-fold in distilled water prior to analysis to minimize multiple scattering. All determinations were conducted in triplicate at 25 °C after an equilibration time of 2 min.

The pH of NE-ZLO-9, toothpaste, and mouthwash formulations was measured at weeks 0, 4, 8, and 12. Before measurement, the samples were equilibrated at room temperature. The pH was determined in triplicate using a calibrated digital pH meter (HI2211 HANNA Basic pH/ORP Benchtop Meter, Hanna Instruments, Bangkok, Thailand) equipped with a glass electrode. Results are expressed as mean ± SD (*n* = 3). The viscosity of NE-ZLO-9 and the toothpaste formulations was evaluated at week 0 and after 12 weeks of storage under all storage conditions, as described in the previous section. The viscosity of the mouthwash formulations was also assessed, but the values were below the measurable range of the instrument under the selected experimental conditions.

### 3.9. UV–Vis Estimation of Apparent Limonene-Equivalent Retention

Changes in the limonene-containing fraction of free ZLO, NE-ZLO-9, and the oral care formulations were monitored using UV–Vis spectrophotometry based on a modified method described by Ahangar and Eslami [39]. A limonene standard (purity ≥95%, Sigma-Aldrich) was prepared in ethanol at concentrations of 10–200 µg/mL, and the calibration curve was constructed from the absorbance measured at 252 nm.

For toothpaste samples, approximately 100 mg was accurately weighed into a microcentrifuge tube and mixed with 3.0 mL of ethanol. The mixture was vortexed for 2 min and centrifuged at 3000 rpm for 15 min to separate the insoluble components. The supernatant was collected and filtered through a 0.45 µm PTFE syringe filter before analysis. Mouthwash samples (100 mg) were mixed with 3.0 mL of ethanol, vortexed for 2 min, and filtered before measurement. Free ZLO (1 µL) and NE-ZLO-9 (10 µL) were diluted with ethanol (3.0 mL) to obtain absorbance values within the calibration range. Blank toothpaste and mouthwash bases (without ZLO or NE-ZLO-9) were treated using the same procedures. Their absorbance values were subtracted from those of the corresponding samples to reduce interference from the formulation excipients.

Absorbance was measured at 252 nm using a Libra S80 UV–Vis spectrophotometer (Biochrom Ltd., Cambridge, UK). The results were expressed as apparent limonene-equivalent concentrations and used only as a preliminary measure of chemical retention.

Apparent retention was calculated using Equation (3):

Apparent retention (%) = (C_t_/C_0_) × 100 (3)

where C_t_ is the apparent limonene-equivalent concentration measured after 4, 8, or 12 weeks of storage, and C_0_ is the corresponding apparent concentration at week 0.

### 3.10. Antibacterial Activity

The antibacterial activity of ZLO and NE-ZLO-9 against *Streptococcus mutans* ATCC 25175 was determined by agar well diffusion and broth microdilution methods according to Clinical and Laboratory Standards Institute guidelines [40,41].

#### 3.10.1. Agar Well Diffusion Assay

*S. mutans* was cultured on Brain Heart Infusion Agar (BHIA) at 37 °C under anaerobic conditions for 24 h. The bacterial suspension was adjusted to a 0.5 McFarland standard (1.5 × 10^8^ CFU/mL) and spread onto BHIA plates. Wells (7 mm in diameter) were made using a sterile cork borer. The samples (100 µL) were added to each well, and the plates were further incubated at 37 °C under anaerobic conditions for 24 h. The inhibition zones were measured in millimeters. The experiment was carried out in duplicate.

#### 3.10.2. Minimum Inhibitory and Bactericidal Concentrations

The MIC and MBC of ZLO and NE-ZLO-9 were determined by the broth microdilution method. Two-fold serial dilutions of the samples were prepared in Brain Heart Infusion Broth (BHIB) in 96-well plates and chlorhexidine was used as a positive control. The bacterial suspension was diluted to 1.0 × 10^6^ CFU/mL and then added to each well. The plates were incubated at 37 °C under anaerobic conditions for 24 h. After incubation, 0.01–0.02% (*w*/*v*) resazurin solution was added to each well, followed by incubation at 37 °C for another 2 h. The lowest concentration with no bacterial growth was recorded as the MIC. Samples from wells with no visible bacterial growth were spread onto agar plates and incubated again. The lowest concentration that showed no bacterial growth after subculture was recorded as the MBC. The experiment was carried out in triplicate.

#### 3.10.3. Bacterial Cell Morphology by Scanning Electron Microscopy (SEM)

The effects of ZLO and NE-ZLO-9 on the morphology of *Streptococcus mutans* ATCC 25175 were examined using scanning electron microscopy (SEM). The bacterial suspension (1.5 × 10^8^ CFU/mL) was treated with ZLO or NE-ZLO-9 at 2 × MIC and incubated at 37 °C for 24 h. After incubation, the cells were collected by centrifugation at 5000 rpm for 5 min and washed three times with phosphate-buffered saline (PBS). The cells were fixed with 2.5% glutaraldehyde in PBS for 2 h and then dehydrated using a series of increasing ethanol concentrations. The samples were dried, mounted on aluminum stubs, coated with gold, and observed using a scanning electron microscope (Quanta 400 FEG; FEI, Hillsboro, OR, USA).

### 3.11. Statistical Analysis

Unless otherwise stated, experiments were performed in triplicate, and the results are expressed as the mean ± standard deviation (SD). Data obtained from the HLB optimization and surfactant concentration optimization studies were analyzed using one-way analysis of variance (ANOVA), followed by Tukey’s multiple comparison test. For the storage stability study, two-way ANOVA followed by Tukey’s multiple comparison test was used to evaluate the effects of storage condition and storage time. Differences were considered statistically significant at *p* < 0.05.

## 4. Conclusions

The present study showed that the combination of shade drying and nanoemulsion formulation is an effective strategy for enhancing the suitability of *Z. limonella* essential oil (ZLO) for oral care applications. Shade drying increased the essential oil yield and altered its chemical profile by increasing the relative abundance of limonene and terpinen-4-ol. The optimized nanoemulsion exhibited a small droplet size and good physical stability, providing better protection of volatile constituents during storage. Nanoencapsulation significantly improved apparent limonene-equivalent retention, particularly under accelerated and continuous light-exposure conditions. Incorporation into toothpaste and mouthwash further enhanced the stability of ZLO; however, the extent of protection depended on the formulation matrix. Toothpaste provided greater inherent protection, potentially associated with its highly viscous matrix, whereas mouthwash benefited more substantially from nanoencapsulation. Overall, these findings demonstrate that nanoemulsification is a promising approach for improving the storage stability of *Z. limonella* essential oil and support its potential application as a stable natural ingredient in oral care products. Although the prototype formulations contained only 0.22% (*w*/*w*) nonionic surfactants, this does not confirm oral mucosal safety, particularly because terpenes may enhance membrane permeability. Therefore, in vitro cytotoxicity and membrane-integrity tests using oral cell models such as TR146 or HGF-1 are still required before further oral healthcare application.

## 5. Patents

The work reported in this manuscript has resulted in a Thai petty patent application entitled “*Zanthoxylum limonella* Essential Oil Nanoemulsion for Oral Care Products and Its Production Process”, Thai Petty Patent Application No. 2603002966, filed on 3 July 2026, which is currently under examination by the Department of Intellectual Property, Thailand.

## Figures and Tables

**Table 1:** Chemical composition of essential oils extracted from fresh (ZLO1) and shade-dried (ZLO2) *Z. limonella* fruits determined by GC–MS analysis.

RT (min)	RI_ref_	RI_cal_		Compounds	Area (%)	
		ZLO1	ZLO2		ZLO1	ZLO2
11.968	930	927	927	*α*-Thujene	0.35	0.72
12.278, 12.296	939	934	934	*α*-Pinene	5.50	2.30
14.089, 14.092	975	974	975	sabinene	11.33	11.02
14.867, 14.857	990	992	992	*β*-Myrcene	3.54	2.16
15.540, 15.498	1002	1007	1006	*α*-Phellandrene	19.75	7.10
15.750	1011	1011	-	*δ*-3-Carene	0.46	-
16.047, 16.054	1017	1017	1018	*α*-Terpinene	0.68	2.30
16.459, 16.462	1024	1026	1026	*p*-Cymene	3.05	3.04
16.724, 16.743	1030	1032	1032	Limonene	29.52	34.84
17.554, 17.567	1050	1049	1050	*cis-β*-Ocimene	5.04	4.74
18.048, 18.070	1059	1060	1060	*γ*-Terpinene	1.21	4.52
18.459	1068	1069	-	*cis*-Sabinene hydrate	0.16	-
18.603	1070	1072	-	*n*-Octanol	0.16	-
19.443, 19.447	1086	1090	1090	Terpinolene	0.47	1.11
20.022, 20.009	1096	1102	1102	Linalool	2.88	1.78
21.025, 21.029	1121	1123	1123	*cis-p*-Menth-2-en-1-ol	0.36	0.63
23.689, 23.764	1177	1180	1182	Terpinen-4-ol	4.44	15.75
24.300, 24.304	1188	1194	1194	*α*-Terpineol	1.64	1.48
24.899, 24.902	1201	1207	1207	*n*-Decanal	1.08	1.16
25.065	1208	-	1210	*trans*-Piperitol	-	0.22
25.157, 25.160	1213	1212	1213	Octyl acetate	0.82	0.97
27.121	1252	1256	1265	Geraniol	0.30	-
27.883	1296	-	1273	*n*-Decanol	-	0.18
28.841	1294	1295	-	2-Undecanone	0.39	-
32.714, 32.694	1381	1386	1385	Geranyl acetate	2.86	0.91
33.744, 33.740	1408	1410	1410	Dodecanal	0.53	0.63
34.322	1419	1425	1425	(E)-Caryophyllene	0.38	0.41
36.823, 36.819	1481	1486	1486	Germacrene D	0.57	0.20

Note: RI_ref_; Kovat Retention Indices refer to Adams, RI_cal_; Kovat Retention Indices according to experimental calculation.

**Table 2:** Effect of surfactant HLB value on the physicochemical characteristics of NE-ZLO.

Formula	Size (nm)	PDI	Zeta Potential (mV)
NE-ZLO-1	62.57 ± 0.98 ^f^	0.222 ± 0.011 ^b^	−24.83 ± 0.72 ^d^
NE-ZLO-2	61.50 ± 0.40 ^f^	0.229 ± 0.002 ^b^	−22.10 ± 0.87 ^e^
NE-ZLO-3	55.67 ± 0.21 ^e^	0.235 ± 0.013 ^b^	−28.27 ± 0.49 ^c^
NE-ZLO-4	51.67 ± 0.45 ^c^	0.217 ± 0.004 ^b^	−29.67 ± 0.42 ^bc^
NE-ZLO-5	49.50 ± 0.10 ^b^	0.174 ± 0.005 ^a^	−31.13 ± 0.76 ^b^
NE-ZLO-6	47.37 ± 0.49 ^a^	0.180 ± 0.015 ^a^	−31.17 ± 0.81 ^b^
NE-ZLO-7	54.20 ± 0.17 ^d^	0.269 ± 0.011 ^c^	−34.93 ± 0.85 ^a^
NE-ZLO-8	64.77 ± 0.25 ^g^	0.322 ± 0.011 ^d^	−36.03 ± 0.64 ^a^

Values are expressed as mean ± SD (*n* = 3). Different lowercase superscripts within the same column indicate statistically significant differences (*p* < 0.05).

**Table 3:** Effect of surfactant concentration on the physicochemical characteristics of ZLO.

Formula	Size (nm)	PDI	Zeta Potential (mV)
NE-ZLO-6	47.37 ± 0.49 ^d^	0.180 ± 0.015 ^a^	−31.17 ± 0.81 ^ab^
NE-ZLO-9	40.83 ± 0.06 ^c^	0.207 ± 0.009 ^ab^	−31.10 ± 0.78 ^ab^
NE-ZLO-10	39.53 ± 0.15 ^b^	0.219 ± 0.009 ^b^	−32.50 ± 0.89 ^a^
NE-ZLO-11	36.23 ± 0.32 ^a^	0.225 ± 0.009 ^b^	−29.70 ± 0.44 ^b^

Values are expressed as mean ± SD (*n* = 3). Different lowercase superscripts within the same column indicate statistically significant differences (*p* < 0.05).

**Table 4:** Changes in pH of NE-ZLO-9 during storage under different conditions.

Storage Time	4 °C	RT	45 °C	Light Exposure
Week 0	6.10 ± 0.01 ^a^			
Week 4	6.06 ± 0.01 ^b,1^	6.06 ± 0.01 ^b,1^	6.06 ± 0.01 ^b,1^	6.06 ± 0.01 ^b,1^
Week 8	5.99 ± 0.02 ^c,1^	5.99 ± 0.02 ^c,1^	5.99 ± 0.02 ^c,1^	5.99 ± 0.02 ^c,1^
Week 12	5.96 ± 0.02 ^c,1^	5.96 ± 0.02 ^c,1^	5.96 ± 0.02 ^c,1^	5.96 ± 0.02 ^c,1^

Values are expressed as mean ± SD (*n* = 3). Different lowercase letters within the same column indicate significant differences among storage times, whereas different superscript numbers within the same row indicate significant differences among storage conditions (*p* < 0.05).

**Table 5:** Changes in particle size of NE-ZLO-9 during storage under different conditions.

Storage Time	4 °C	RT	45 °C	Light Exposure
Week 0	40.83 ± 0.06 ^c,1^	40.83 ± 0.06 ^a,1^	40.83 ± 0.06 ^d,1^	40.83 ± 0.06 ^a,1^
Week 4	38.13 ± 0.29 ^b,2^	59.47 ± 0.15 ^b,3^	35.07 ± 0.12 ^c,1^	61.97 ± 0.21 ^b,4^
Week 8	37.57 ± 0.06 ^a,2^	73.50 ± 0.17 ^c,3^	34.17 ± 0.29 ^b,1^	73.37 ± 0.51 ^c,3^
Week 12	37.50 ± 0.20 ^a,2^	79.27 ± 0.31 ^d,3^	32.17 ± 0.15 ^a,1^	99.13 ± 0.06 ^d,4^

Values are presented as mean ± SD (*n* = 3). Different lowercase letters within the same column indicate significant differences among storage times, whereas different superscript numbers within the same row indicate significant differences among storage conditions (*p* < 0.05).

**Table 6:** Changes in polydispersity index (PDI) of NE-ZLO-9 during storage under different conditions.

Storage Time	4 °C	RT	45 °C	Light Exposure
Week 0	0.211 ± 0.009 ^b,1^	0.211 ± 0.009 ^b,1^	0.211 ± 0.009 ^c,1^	0.211 ± 0.009 ^a,1^
Week 4	0.182 ± 0.001 ^a,2^	0.225 ± 0.001 ^b,3^	0.132 ± 0.002 ^b,1^	0.224 ± 0.004 ^a,3^
Week 8	0.175 ± 0.000 ^a,2^	0.216 ± 0.004 ^b,3^	0.104 ± 0.002 ^a,1^	0.218 ± 0.008 ^a,3^
Week 12	0.186 ± 0.007 ^a,2^	0.187 ± 0.002 ^a,2^	0.105 ± 0.002 ^a,1^	0.343 ± 0.006 ^b,3^

Values are presented as mean ± SD (*n* = 3). Different lowercase letters within the same column indicate significant differences among storage times, whereas different superscript numbers within the same row indicate significant differences among storage conditions (*p* < 0.05).

**Table 7:** Changes in zeta potential of NE-ZLO-9 during storage under different conditions.

Storage Time	4 °C	RT	45 °C	Light Exposure
Week 0	−31.10 ± 0.78 ^a,1^	−31.10 ± 0.78 ^a,1^	−31.10 ± 0.78 ^a,1^	−31.10 ± 0.78 ^a,1^
Week 4	−30.93 ± 0.95 ^a,1^	−30.90 ± 0.20 ^a,1^	−30.40 ± 0.26 ^a,1^	−30.60 ± 0.95 ^a,1^
Week 8	−30.70 ± 0.44 ^a,1^	−30.83 ± 0.47 ^a,1^	−27.10 ± 0.78 ^b,2^	−30.00 ± 0.92 ^a,1^
Week 12	−30.67 ± 0.45 ^a,1^	−30.13 ± 0.67 ^a,1^	−23.77 ± 0.64 ^c,2^	−29.63 ± 0.59 ^a,1^

Values are presented as mean ± SD (*n* = 3). Different lowercase letters within the same column indicate significant differences among storage times, whereas different superscript numbers within the same row indicate significant differences among storage conditions (*p* < 0.05).

**Table 8:** Limonene-equivalent retention (%) in free ZLO and NE-ZLO-9 during storage under different conditions.

Sample	W4	W8	W12
ZLO at 4 °C	68.73 ± 0.78 ^a^	58.53 ± 1.46 ^a^	49.71 ± 1.09 ^a^
ZLO at RT	68.57 ± 0.46 ^a^	57.33 ± 0.07 ^a^	46.30 ± 0.64 ^a^
ZLO at 45 °C	56.85 ± 1.13 ^c^	33.16 ± 0.86 ^c^	21.98 ± 0.47 ^c^
ZLO at Light	64.91 ± 0.35 ^b^	41.65 ± 0.48 ^b^	28.99 ± 1.61 ^b^
NE-ZLO-9 at 4 °C	93.74 ± 1.54 ^A^*	90.36 ± 0.54 ^A^*	87.65 ± 0.49 ^A^*
NE-ZLO-9 at RT	91.89 ± 1.41 ^A^*	88.07 ± 0.54 ^A^*	85.06 ± 0.60 ^A^*
NE-ZLO-9 at 45 °C	75.86 ± 1.22 ^C^*	66.26 ± 0.52 ^C^*	58.84 ± 0.68 ^C^*
NE-ZLO-9 at Light	88.15 ± 1.03 ^B^*	83.09 ± 1.22 ^B^*	78.94 ± 1.27 ^B^*

Values are presented as mean ± SD (*n* = 3). Different lowercase and uppercase letters indicate significant differences among storage conditions within free ZLO and NE-ZLO-9, respectively (*p* < 0.05). Asterisks indicate significant differences between free ZLO and NE-ZLO-9 under the same condition (*p* < 0.05).

**Table 9:** Viscosity of toothpaste formulations containing free ZLO and NE-ZLO-9 during storage.

Sample	W0 (×10^3^ cP)	W12 (×10^3^ cP)	ΔViscosity (W12 − W0) (×10^3^ cP)
TP-ZLO at 4 °C	35.91 ± 0.01	33.13 ± 0.03	−2.78
TP-ZLO at RT		36.44 ± 0.02	+0.53
TP-ZLO at 45 °C		43.64 ± 0.02	+7.73
TP-ZLO at Light		35.72 ± 0.03	−0.19
TP-NE-ZLO-9 at 4 °C	34.84 ± 0.01	32.04 ± 0.01	−2.80
TP-NE-ZLO-9 at RT		33.12 ± 0.01	−1.72
TP-NE-ZLO-9 at 45 °C		45.43 ± 0.03	+10.59
TP-NE-ZLO-9 at Light		34.82 ± 0.03	−0.02

**Table 10:** pH of toothpaste formulations containing free ZLO and NE-ZLO-9 during storage.

Sample	W0	W4	W8	W12	ΔpH
TP-ZLO at 4 °C	6.90 ± 0.02 *	6.70 ± 0.01 ^a^	6.66 ± 0.01 ^a^	6.63 ± 0.01 ^a^	−0.27
TP-ZLO at RT		6.68 ± 0.02 ^ab^	6.62 ± 0.02 ^ab^	6.58 ± 0.02 ^b^	−0.32
TP-ZLO at 45 °C		6.68 ± 0.01 ^ab^	6.60 ± 0.02 ^b^	6.52 ± 0.01 ^c^	−0.38
TP-ZLO at Light		6.65 ± 0.02 ^c^*	6.55 ± 0.02 ^c^*	6.46 ± 0.02 ^d^*	−0.44
TP-NE-ZLO-9 at 4 °C	6.65 ± 0.02	6.70 ± 0.02 ^AB^	6.68 ± 0.02 ^A^	6.66 ± 0.02 ^A^	+0.01
TP-NE-ZLO-9 at RT		6.66 ± 0.02 ^B^	6.63 ± 0.02 ^B^	6.60 ± 0.02 ^B^	−0.05
TP-NE-ZLO-9 at 45 °C		6.73 ± 0.01 ^A^*	6.66 ± 0.01 ^AB^*	6.58 ± 0.02 ^B^*	−0.07
TP-NE-ZLO-9 at Light		6.51 ± 0.02 ^C^	6.45 ± 0.00 ^C^	6.38 ± 0.02 ^C^	−0.27

Values are presented as mean ± SD (*n* = 3). Different lowercase and uppercase letters indicate significant differences among storage conditions within free ZLO and NE-ZLO-9, respectively (*p* < 0.05). Asterisks indicate significant differences between free ZLO and NE-ZLO-9 under the same condition (*p* < 0.05).

**Table 11:** pH of mouthwash formulations containing free ZLO and NE-ZLO-9 during storage.

Sample	W0	W4	W8	W12	ΔpH
MW-ZLO at 4 °C	6.85 ± 0.02 *	6.80 ± 0.02 ^a^*	6.72 ± 0.00 ^a^*	6.35 ± 0.02 ^b^	−0.13
MW-ZLO at RT		6.72 ± 0.01 ^b^*	6.55 ± 0.01 ^b^	6.42 ± 0.02 ^a^	−0.43
MW-ZLO at 45 °C		6.71 ± 0.02 ^b^*	6.56 ± 0.00 ^b^*	6.40 ± 0.02 ^a^	−0.45
MW-ZLO at Light		6.65 ± 0.01 ^c^*	6.45 ± 0.01 ^c^*	6.30 ± 0.01 ^c^	−0.55
MW-NE-ZLO-9 at 4 °C	6.79 ± 0.02	6.69 ± 0.02 ^A^	6.64 ± 0.02 ^A^	6.60 ± 0.02 ^A^*	−0.15
MW-NE-ZLO-9 at RT		6.63 ± 0.01 ^B^	6.58 ± 0.02 ^B^	6.52 ± 0.01 ^B^*	−0.27
MW-NE-ZLO-9 at 45 °C		6.59 ± 0.02 ^B^	6.50 ± 0.02 ^C^	6.42 ± 0.02 ^C^	−0.37
MW-NE-ZLO-9 at Light		6.50 ± 0.02 ^C^	6.38 ± 0.02 ^D^	6.28 ± 0.01 ^D^	−0.51

Values are presented as mean ± SD (*n* = 3). Different lowercase and uppercase letters indicate significant differences among storage conditions within free ZLO and NE-ZLO-9, respectively (*p* < 0.05). Asterisks indicate significant differences between free ZLO and NE-ZLO-9 under the same condition (*p* < 0.05).

**Table 12:** Limonene-equivalent retention (%) in toothpaste formulations containing free ZLO and NE-ZLO-9 during storage.

Sample	W4	W8	W12
TP-ZLO at 4 °C	82.10 ± 1.12 ^a^	76.41 ± 1.97 ^a^	71.59 ± 0.96 ^a^
TP-ZLO at RT	79.97 ± 0.92 ^a^	74.48 ± 0.90 ^a^	69.77 ± 1.18 ^a^
TP-ZLO at 45 °C	73.03 ± 0.72 ^b^	67.25 ± 1.54 ^b^	62.92 ± 0.55 ^b^
TP-ZLO at Light	76.67 ± 0.77 ^b^	70.21 ± 1.38 ^b^	63.81 ± 1.16 ^b^
TP-NE-ZLO-9 at 4 °C	89.80 ± 1.08 ^A^*	83.34 ± 1.65 ^A^*	77.62 ± 1.21 ^A^*
TP-NE-ZLO-9 at RT	86.99 ± 0.92 ^A^*	80.43 ± 1.87 ^A^*	73.67 ± 1.74 ^A^*
TP-NE-ZLO-9 at 45 °C	80.00 ± 1.81 ^B^*	71.28 ± 1.42 ^B^*	69.24 ± 0.84 ^B^*
TP-NE-ZLO-9 at Light	79.78 ± 0.94 ^B^	73.43 ± 2.77 ^B^	69.42 ± 0.58 ^B^*

Values are presented as mean ± SD (*n* = 3). Different lowercase and uppercase letters indicate significant differences among storage conditions within free ZLO and NE-ZLO-9, respectively (*p* < 0.05). Asterisks indicate significant differences between free ZLO and NE-ZLO-9 under the same condition (*p* < 0.05).

**Table 13:** Limonene-equivalent retention (%) in mouthwash formulations containing free ZLO and NE-ZLO-9 during storage.

Sample	W4	W8	W12
MW-ZLO at 4 °C	82.55 ± 1.15 ^a^	77.05 ± 2.29 ^a^	71.78 ± 1.27 ^a^
MW-ZLO at RT	80.25 ± 1.11 ^a^	74.60 ± 1.24 ^a^	69.55 ± 1.58 ^a^
MW-ZLO at 45 °C	73.05 ± 1.02 ^b^	68.80 ± 0.96 ^b^	62.82 ± 0.74 ^b^
MW-ZLO at Light	76.73 ± 1.08 ^b^	70.20 ± 1.95 ^b^	64.14 ± 1.42 ^b^
MW-NE-ZLO-9 at 4 °C	90.14 ± 1.29 ^A^*	83.44 ± 2.32 ^A^*	77.44 ± 1.65 ^A^*
MW-NE-ZLO-9 at RT	86.83 ± 1.24 ^A^*	80.53 ± 2.62 ^A^*	73.95 ± 2.37 ^A^*
MW-NE-ZLO-9 at 45 °C	80.93 ± 1.13 ^B^*	75.37 ± 3.55 ^B^*	69.25 ± 1.18 ^B^*
MW-NE-ZLO-9 at Light	80.06 ± 1.13 ^B^*	72.53 ± 3.24 ^B^*	67.72 ± 1.90 ^B^*

Values are presented as mean ± SD (*n* = 3). Different lowercase and uppercase letters indicate significant differences among storage conditions within free ZLO and NE-ZLO-9, respectively (*p* < 0.05). Asterisks indicate significant differences between free ZLO and NE-ZLO-9 under the same condition (*p* < 0.05).

**Table 14:** MIC and MBC of samples and controls against *S. mutans*.

Test Substance	MIC	MBC
**Samples**		
NE-ZLO-9	3.125%	>50%
ZLO	0.078%	0.3125%
**Controls**		
DMSO	11.25%	>22.5%
Chlorhexidine	0.0625 µg/mL	0.5 µg/mL

Values are expressed as percentage (% *v*/*v*) for samples and DMSO, and µg/mL for chlorhexidine. MIC = minimum inhibitory concentration; MBC = minimum bactericidal concentration.

**Table 15:** Composition of nanoemulsion formulations used for HLB optimization and surfactant concentration optimization.

Ingredient	NE-ZLO-1	NE-ZLO-2	NE-ZLO-3	NE-ZLO-4	NE-ZLO-5	NE-ZLO-6	NE-ZLO-7	NE-ZLO-8	NE-ZLO-9	NE-ZLO-10	NE-ZLO-11
HLB	10.0	11.0	12.0	13.0	13.5	14.0	14.5	15.0	14.0	14.0	14.0
ZLO-2	10	10	10	10	10	10	10	10	10	10	10
Jojoba oil	10	10	10	10	10	10	10	10	10	10	10
Span 80	9.35	7.48	5.61	3.74	2.80	1.86	0.94	0.00	2.06	2.24	2.43
PEG-40 HCO	5.33	6.26	7.20	8.14	8.60	9.07	9.53	10.0	9.97	10.88	11.78
Tween 80	5.33	6.26	7.20	8.14	8.60	9.07	9.53	10.0	9.97	10.88	11.78
Glycerin	10	10	10	10	10	10	10	10	10	10	10
Water	50	50	50	50	50	50	50	50	48	46	44
**Total**	**100**	**100**	**100**	**100**	**100**	**100**	**100**	**100**	**100**	**100**	**100**

**Table 16:** Quantitative compositions of toothpaste formulations containing free ZLO or NE-ZLO-9.

Ingredient	TP-ZLO (% *w*/*w*)	TP-NE-ZLO-9 (% *w*/*w*)
Sodium carboxymethyl cellulose	1.30	1.30
Xanthan gum	1.20	1.20
Propylene glycol	4.50	4.50
Deionized water	37.51	37.51
Sodium benzoate	0.45	0.45
Sodium laureth sulfate (Texapon N8000)	8.74	8.74
Glycerin	13.00	13.00
Hydrated silica	15.00	15.00
Hydroxyapatite	2.00	2.00
Titanium dioxide	0.30	0.30
Calcium carbonate	15.00	15.00
Tween 80	0.45	–
PEG-40 hydrogenated castor oil	0.45	–
Free ZLO	0.10	–
NE-ZLO-9	–	1.00
**Total**	**100.00**	**100.00**

**Table 17:** Quantitative compositions of mouthwash formulations containing free ZLO or NE-ZLO-9.

Ingredient	MW-ZLO (% *w*/*w*)	MW-NE-ZLO-9 (% *w*/*w*)
Deionized water	85.80	85.20
Sodium benzoate	0.70	0.70
Glycerin	12.00	12.00
Sodium chloride	0.50	0.50
Tween 80	0.45	-
PEG-40 hydrogenated castor oil	0.45	-
Free ZLO	0.10	-
NE-ZLO-9	-	1.00
**Total**	**100.00**	**100.00**

## Data Availability

The data presented in this study are available from the corresponding author upon reasonable request.

## Abbreviations

The following abbreviations are used in this manuscript:

DLS	Dynamic Light Scattering
GC–MS	Gas Chromatography–Mass Spectrometry
HLB	Hydrophilic–Lipophilic Balance
MW	Mouthwash
NE-ZLO	*Zanthoxylum limonella* Essential Oil Nanoemulsion
O/W	Oil-in-Water
PDI	Polydispersity Index
RI	Retention Index
RT	Room Temperature
SD	Standard Deviation
TP	Toothpaste
UV–Vis	Ultraviolet–Visible Spectrophotometry
ZLO	*Zanthoxylum limonella* Essential Oil
